# Multi-Field CEST NMR Reconstructs a Protein Folding Landscape

**DOI:** 10.1021/jacs.6c16569

**Published:** 2026-10-02

**Authors:** Ved Prakash Tiwari, Laetitia Rožić, Sebastian Hiller

**Affiliations:** 27209 Biozentrum, University of Basel, Klingelbergstrasse 70, Basel 4056, Switzerland

## Abstract

Protein dynamics are central to their folding and function, yet the sparsely populated conformational states that underpin these processes remain difficult to characterize at atomic resolution. Chemical exchange saturation transfer (CEST) NMR can detect such states and is particularly sensitive to rapid exchange between two minor conformations. This “minor-state exchange”, however, broadens and displaces the minor-state dip in CEST profiles, leading to parameter degeneracy that can preclude quantitative reconstruction of multistate exchange networks. Here, we show that, in the presence of rapid “minor-exchange”, the apparent minor-state chemical shift obtained from two-state fits varies systematically with the static magnetic field, *B*_0_. We exploit this field dependence to develop a nonperturbative, multi-field CEST strategy that distinguishes otherwise degenerate exchange models. Applying multi-field ^15^N-CEST to the model protein Im7 revealed a folding intermediate populated at ∼1% that exchanges with the folded state on a ∼2 ms time scale and with an unfolded state populated at ∼0.4% on a ∼200 μs time scale. Although the unfolded state does not produce a separate CEST dip, its exchange is encoded in the line width and *B*_0_-dependent position of the intermediate-state dip. Analysis of the ^15^N-CEST data provides populations, microscopic exchange rates, and residue-specific ^15^N chemical shifts for all three states. The resulting Im7 free-energy landscape is sequential, with folding proceeding through the intermediate and no detectable direct exchange between the folded and unfolded states. Multi-field CEST provides a broadly applicable, perturbation-free approach for resolving biomolecular dynamics at equilibrium.

## Graphical abstract

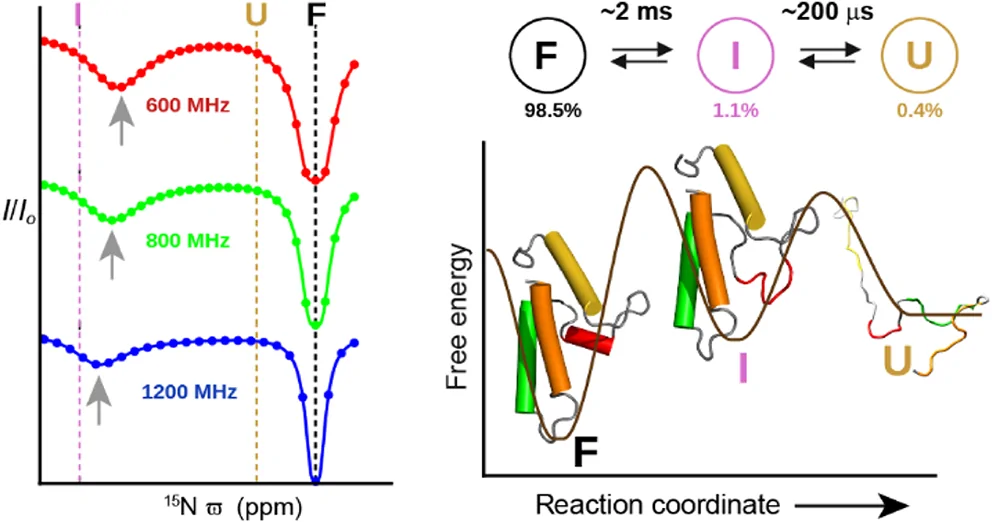

## Introduction

Proteins are inherently dynamic molecules that populate conformational ensembles including a ground state as well as additional high energy conformational states.^1–3^ The populations of these high-energy, minor states and the rates at which they interconvert are governed by the underlying free-energy surface.^4–6^ Despite their sparse population and short lifetime, the minor states often play important roles in protein function, misfunction, misfolding, and folding and their detection and characterization at the atomic level is therefore essential.^7–14^

Exchange-based NMR methods, such as Carr–Purcell–Meiboom–Gill (CPMG) relaxation dispersion, chemical exchange saturation transfer (CEST), and the related dark-state exchange saturation transfer (DEST), detect low-populated states through their exchange effects on observable ground-state signal.^15–19^ These experiments provide state populations, interconversion rates, and atomistic structural information in the form of chemical shifts, for states populated at levels as low as ∼0.5%.^20–24^ Because the sparsely populated protein states remain invisible to most other biophysical approaches, NMR spectroscopy remains critical for their study.^21,25–27^

CPMG relaxation-dispersion is particularly sensitive to microsecond-to-millisecond exchange and has consequently been widely used to characterize excited conformations.^13,14,16,20,26,28^ However, when more than one minor state is populated and exchange between these minor states (“minor-exchange”) is fast, CPMG profiles often remain consistent with a simple two-state exchange model, and the additional minor state escapes detection.^24,29–32^ For such multi-state exchange processes, CEST experiments have become increasingly popular.^24,29,30,33,34^ In a CEST experiment, a weak *B*_1_ field is applied over a series of frequency offsets, while the intensity of the observable major-state resonance is monitored.^17,23^ Saturation at the resonance frequency of the major state produces a strong reduction in peak intensity, whereas saturation at the resonance frequency of the exchanging, low-populated minor state(s) is transferred to the major state through chemical exchange and gives rise to smaller dip(s) in the peak intensity profile at the chemical shift positions of otherwise invisible minor state(s).^17,23,35^

Though CEST experiments were initially devised to study slow exchange processes,^17,23,35^ it was recently shown that they are extremely sensitive to fast exchange between minor states.^24,29,30,33^ In case of fast “minor exchange”, the apparent minor-state dip in the CEST profiles becomes broadened and shifted from the true resonance position of either minor state.^24,29,30^ Extraction of accurate state-specific populations and chemical shifts is complicated, as the exchange-induced broadening depends on the magnitude, but not the sign, of the chemical shift difference between the two minor states, leading to a plethora of possible solutions.^29,30^ For example, for N residues there exist 2^N^ degenerate solutions.^29^ One strategy to resolve this ambiguity is to alter the relative populations of the minor states—for example by variation of the temperature—and to monitor the resulting displacement of the apparent minor state dip.^29,30^ Importantly, however, changing the sample conditions readily alters the free energy surface itself, including the introduction of additional conformational states that can preclude the analysis.^24,30,36–38^ For many systems, changing physiological conditions is thus not experimentally feasible without compromising protein stability, folding behavior, or biological relevance.^39–42^ A strategy is therefore highly needed in which a multi-state ensemble is interrogated under unperturbed solution conditions.

Here, we show that the magnetic-field dependence of minor-state dip position in CEST profiles provides such an approach. In case of ms-μs exchange between two minor states, the direction of the *B*_0_-dependent minor dip displacement allows for the complete and unambiguous determination of the chemical shift positions of the exchanging minor states. We applied this approach to resolve the equilibrium folding landscape of the colicin E7 immunity protein Im7. Im7 is an 87-residue four-helix protein and a benchmark for non-two-state folding.^36,43–46^ Despite extensive studies, the native-condition folding landscape of Im7 has remained incomplete because the unfolded state could never be detected directly at equilibrium.^45,47–49^ Multi-field ^15^N CEST now reveals the unfolded state, defines an effectively sequential F↔I↔U exchange network, and provides residue-specific chemical shifts and microscopic rates for reconstructing the Im7 free-energy landscape from a single equilibrium experiment.

## Results

### Field-Dependence of CEST Profiles Reports on Fast Exchange between Minor States

We first consider how exchange between two minor states affects the position and line width of the apparent minor-state dip in the CEST profiles. Although CEST profiles are described by the full Bloch–McConnell equations in the presence of an offset-dependent saturation (*B*_1_) field,^17,50^ the exchange-induced displacement and broadening of the minor-state dips in the CEST profiles can be intuitively understood using the simpler free-precession Bloch–McConnell equations for the two exchanging minor states.^50,51^ For an isolated spin undergoing a three-state exchange process $A\underset{k_{AB}}{\overset{k_{BA}}{⇋}}B\underset{k_{BC}}{\overset{k_{CB}}{⇋}C}$ , with *p*_*A*_ ≫ *p*_*B*_ ≫ *p*_*C*_, and *k*_*ex*,*BC*_ ≫ *k*_*ex*,*AB*_, where *p*_*i*_ is the population of state *i* and *k*_*ex,ij*_ = *k*_*ij*_ + *k*_*ji*_, the effect of B↔C exchange on state B resonance can be understood by the Bloch–McConnell equations for the evolution of transverse magnetization (*M*_+_ = *M*_*x*_ + *iM*_*y*_) components from states B and C, under free precession:

1 $$\frac{d}{dt}\left[\begin{array}{l} M_{+B}\\ M_{+C} \end{array}\right]=\left[\begin{array}{ll} -R_{2}-k_{BC}-i\omega_{B} & k_{CB}\\ k_{BC} & -R_{2}-k_{CB}-i\omega_{C} \end{array}\right]\left[\begin{array}{l} M_{+B}\\ M_{+C} \end{array}\right]$$

Here, *ω*_*i*_ is the resonance frequency of state *i* in radian/second and *R*_2_ is the transverse relaxation rate, assumed to be the same in all states. For simplicity, we have ignored the effect of $A\underset{k_{AB}}{\overset{k_{BA}}{⇋}}B$ exchange on the B state resonance. Because chemical exchange mixes the magnetization of states B and C, the observed minor-state dip in the CEST profile is not a pure B-state resonance. Instead, the B↔C exchange broadens this dip and shifts its position away from the intrinsic B state chemical shift. Equation 1 has been solved previously^52–54^ and approximate expressions for the exchange-induced shift and exchange-induced line broadening of B state resonance are given by

2 $$\delta_{ex}=\frac{k_{BC}k_{CB}\left(\omega_{C}-\omega_{B}\right)}{\left(k_{CB}^{2}+{\left(\omega_{C}-\omega_{B}\right)}^{2}\right)}=\frac{k_{BC}k_{CB}2\pi B_{0}\left(\varpi_{C}-\varpi_{B}\right)}{(k_{CB}^{2}+\left(2\pi B_{0}\left(\varpi_{C}-\varpi_{B}\right){)}^{2}\right)}$$

3 $$R_{ex}=\frac{k_{BC}{\left(\omega_{C}-\omega_{B}\right)}^{2}}{\left(k_{CB}^{2}+{\left(\omega_{C}-\omega_{B}\right)}^{2}\right)}=\frac{k_{BC}{\left(2\pi B_{0}(\varpi_{C}-\varpi_{B})\right)}^{2}}{(k_{CB}^{2}+\left(2\pi B_{0}\left(\varpi_{C}-\varpi_{B}\right){)}^{2}\right)}$$

Here, *ϖ*_*i*_ is the resonance frequency of state *i* in ppm and *B*_0_ is the magnetic field strength in MHz. Equation 3 shows that broadening of the B-state resonance is proportional to the square of the chemical shift difference and consequently insensitive to the sign of Δϖ_*BC*_ (ϖ_*C*_ – ϖ_*B*_). The broadening alone thus cannot establish whether state C lies upfield or downfield of state B. Equation 2, by contrast, shows that the exchange induced shift in B-state resonance position depends on both the sign and magnitude of Δϖ_*BC*_. From eq 2 it also follows that the magnitude of the exchange-induced shift, expressed in ppm, $\left(\frac{\delta_{ex}}{2\pi B_{0}}\right)$ , decreases with increasing *B*_0_, from a plateau equal to $\frac{k_{BC}\left(\varpi_{C}-\varpi_{B}\right)}{k_{CB}}$ in the fast-exchange limit, *k*_*CB*_ ≫ |ω_*C*_ – ω_*B*_|, towards zero in the slow-exchange limit, *k*_*CB*_ ≪ |ω_*C*_ – ω_*B*_|,^53^ where the two states would give rise to separate dips. This is because increasing *B*_0_ makes the B↔C exchange process slower on the NMR chemical shift time scale, since the frequency difference (ω_*C*_ – ω_*B*_) grows linearly with *B*_0_ while *k*_*CB*_ does not. As a consequence, the two resonances are averaged less completely, and the observed minor-state dip moves back toward the intrinsic B-state chemical shift. The exchange-induced broadening in eq 3 shows the opposite trend, increasing with *B*_0_ and approaching *k_BC_* in the slow-exchange limit.^53^

To illustrate this effect, we have simulated ^15^N CEST profiles for a two-state A↔B exchange process (Figure 1A) and for two three-state A↔B↔C exchange processes, in which state C either lies upfield (Figure 1B) or downfield (Figure 1C) with respect to state B. In case of two-state exchange, neither Δϖ_2*st*_ nor Δ*R*_2_ (= *R*_2*B*_ – *R*_2*A*_), which is akin to *R*_*ex*_, change with *B*_0_ (Figure 1A). In contrast, in the presence of the additional B↔C exchange, the minor-state dip is strongly broadened (Figure 1B, C) and its position becomes *B*_0_-dependent. As predicted by eqs 2 and 3, the direction of the field-dependent shift depends on the sign of Δϖ_*BC*_, whereas the exchange-induced broadening Δ*R*_2_ does not (Figure 1B, C). Consistent with the field dependence of *δ*_*ex*_ described above, the apparent minor-state dip moves toward the true B-state chemical shift as *B*_0_ increases. These simulations therefore establish that the chemical shift position of the C-state, which is itself invisible in the CEST profiles, can be obtained from the *B*_0_-dependence of the minor-state dip. Importantly, this effect is experimentally accessible because the minor-state dip position in ^15^N CEST profiles can be determined with high precision,^17,55,56^ with estimated accuracies of 20 ppb at 600 MHz, 15 ppb at 800 MHz, and 10 ppb at 1200 MHz (Supplementary Figure S1) for |Δϖ_2*st*_| > 2 ppm. Thus, multi-field CEST measurements provide a practical route to detecting hidden exchange between minor states and to constraining otherwise ambiguous three-state exchange models.

**Figure 1:**
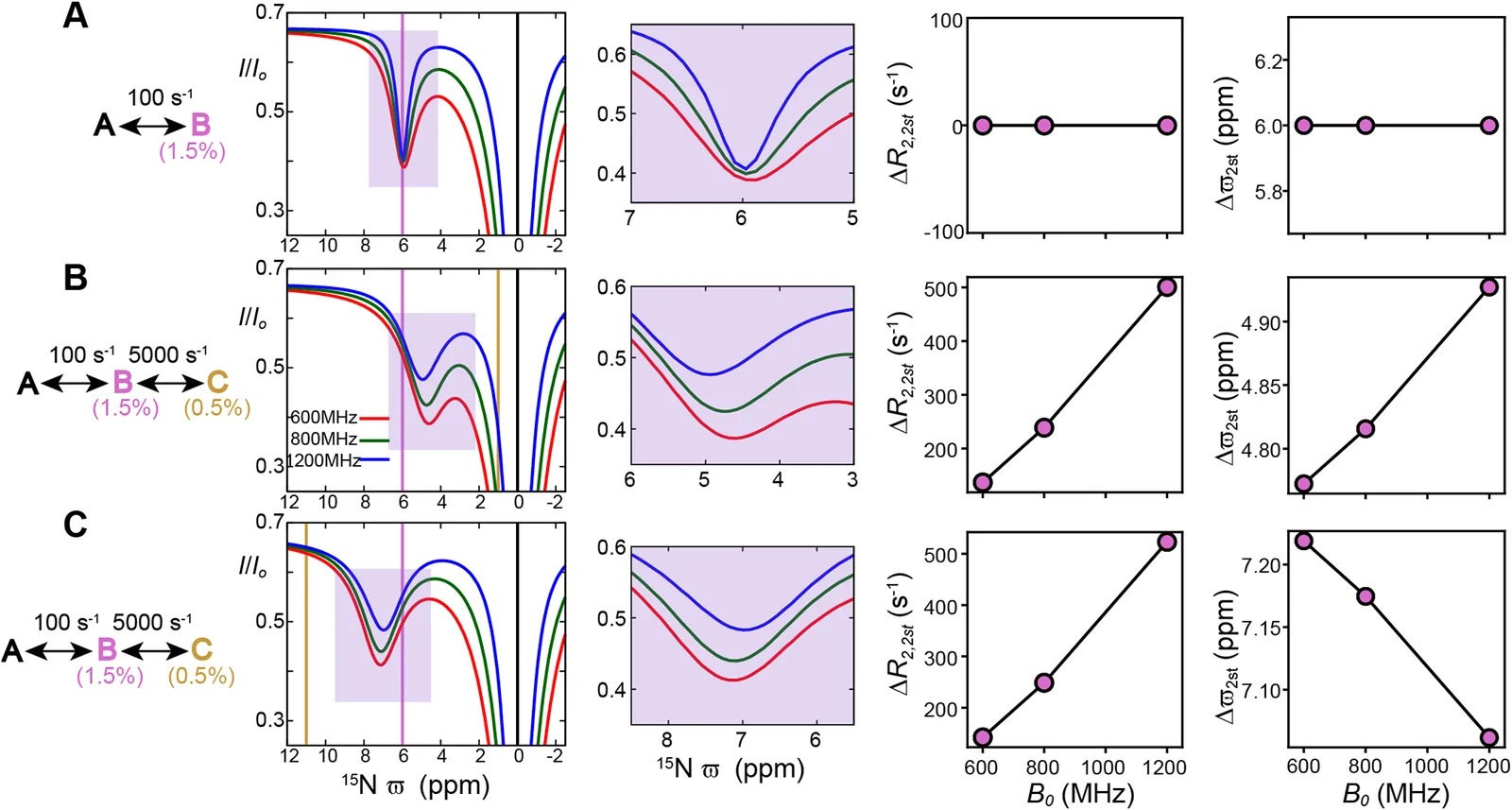
Dependence of the minor-state CEST dip on the *B*_0_-field. A) ^15^N CEST profiles simulated at three *B*_0_ field strengths: 600 MHz (red), 800 MHz (green), and 1200 MHz (blue) using *B*_1_ = 35 Hz. ϖ_*A*_ = 0 ppm, ϖ_*B*_ = 6 ppm. The exchange scheme is shown on the left, and the shaded region containing the minor-state dip is enlarged in the adjacent panel. ΔR_2,2*st*_ and Δϖ_2*st*_, obtained from the two-state fits to the data are plotted as a function of *B*_0_ field strength on the right. For A↔B exchange, both parameters remain independent of *B*_0_. B) Corresponding simulations for a three-state A↔B↔C exchange process in which state C lies upfield of state B (ϖ_*C*_ = 1 ppm). C) Corresponding simulations for a three-state A↔B↔C exchange process in which state C lies downfield of state B (ϖ_*C*_ = 11 ppm). Note that the *B*_0_-dependence of Δϖ_2*st*_ occurs in the opposite direction in (C) relative to (B).

### Im7 Populates More Than Two Conformations at 10 °C

We applied this approach to the folding dynamics of the colicin E7 immunity protein Im7. Early stopped-flow experiments showed that Im7 does not fold by a simple two-state mechanism, but instead populates a folding intermediate.^36,43,57^ Subsequent relaxation-dispersion NMR studies showed that, under native equilibrium conditions at 10 °C, this intermediate is populated at less than 2% and exchanges with the folded state on the millisecond time scale.^45,47,48,58^ These properties make Im7 well suited for CEST NMR experiments. We therefore recorded ^15^N CEST profiles of [*U*-^15^N, ^1^H] WT Im7 at 10 °C and 800 MHz proton frequency. The 2D ^15^N–^1^H correlation spectrum of Im7 under these conditions is well resolved, allowing exchange to be studied for most backbone amide sites (Figure 2A). Several residues displayed a minor dip in their ^15^N CEST profiles in addition to the major dip arising from the native ground state (Figure 2B). For the initial analysis, we selected a set of 19 residues, referred to as set19, for which the magnitude of the apparent two-state chemical shift difference, |Δϖ_2*st*_|, was greater than 2 ppm (see Supplementary Information). The chemical shifts of these minor-state dips, obtained from two-state fits of the data, agreed well with the reported chemical shifts of the double mutant L53A/I54A Im7 (Figure 2C). This double mutant had previously been designed to truncate helix-III side chains, thus selectively destabilizing the folded state F and populating the intermediate I.^45,59^ This match confirms that the CEST profiles are indeed probing the F↔I exchange process.

**Figure 2:**
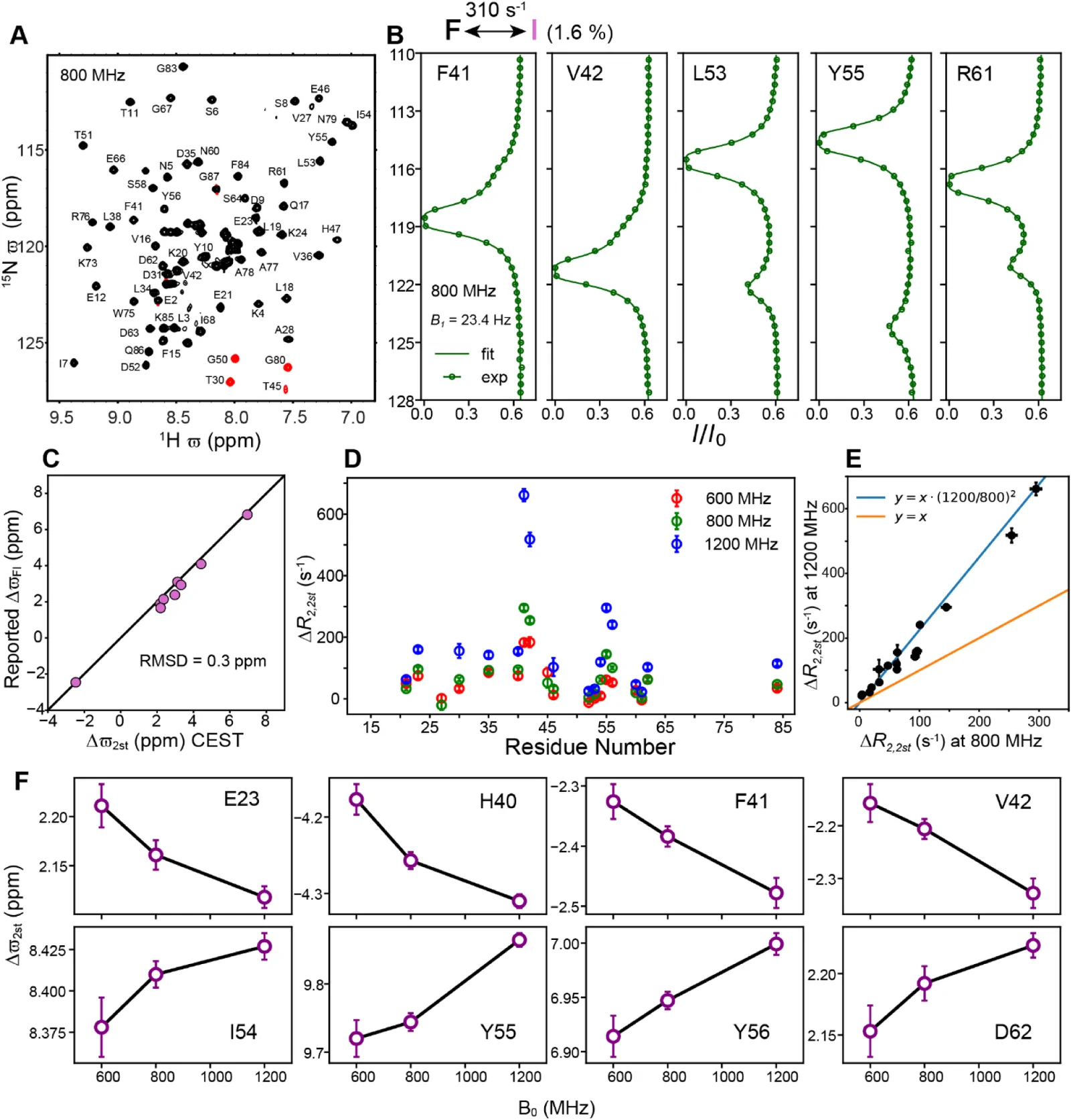
^15^N CEST of Im7. A) ^15^N–^1^H correlation spectrum of WT Im7 recorded at 10 °C and 800 MHz. Sequence-specific resonance assignments are indicated. B) Representative ^15^N CEST profiles of five residues. Experimental data points are shown as green circles, and the green lines represent the best fits to the experimental data. The best-fit exchange model is shown above the profiles. C) Minor-state chemical shifts obtained from two-state fits of the CEST data are compared with the chemical shifts of the known Im7 folding intermediate.^44^ Δϖ_2*st*_ denotes the apparent chemical shift difference between the major and minor states obtained from the ^15^N CEST profiles, whereas reported Δϖ*_FI_* was calculated as ϖ_*F*,*L*53A/*I*54A–*Im*7_ – ϖ_*F*,*WT*–*Im*7_. Here, ϖ_*F*,*L*53A/*I*54A–*Im*7_ denotes the ground-state chemical shifts of the L53A/I54A-Im7 variant, which mimics the folding intermediate state,^44^ and ϖ_*F*,*WT*–*Im*7_ denotes the ground-state chemical shifts of WT Im7 used in the present study. Values are shown for the nine residues of set19 for which ϖ_*F*,*L*53A/*I*54A–*Im*7_ has been reported; residues at and immediately adjacent to the substitution sites are excluded.^44^ D) Apparent ΔR_2,2*st*_ values, defined as *R*_2,_ *_I_* – *R*_2,*F*_, for residues with |Δϖ_2*st*_| > 2 ppm, obtained from two-state fits of the CEST data at 600 MHz (red), 800 MHz (green), and 1200 MHz (blue). E) Comparison of ΔR_2,2*st*_ values obtained at 1200 and 800 MHz. F) *B*_0_-dependent variation of Δϖ_2*st*_ for eight residues showing a monotonic change in Δϖ_2*st*_ with *B*_0_ field strength.

We next asked whether the CEST data could be described by a two-state exchange model, or whether a more complex exchange process had to be considered. We first fitted ^15^N CEST data sets recorded at 800 MHz with *B*_1_ fields of 23.4 and 58.6 Hz (Supplementary Table S1) using a two-state exchange model F↔I under the *R*_2_ *_I_* = *R*_2*F*_ constraint. This constraint is physically meaningful as the Im7 intermediate is expected to be compact.^60,61^ However, this model did not adequately reproduce the data, giving $\chi_{red}^{2}=2.7$ (Figure S2). In contrast, allowing *R*_2*I*_ to vary independently resulted in good-quality fits ( $\chi_{red}^{2}=1.0$ ; Figure 2B) with *k*_*ex*,*FI*_ = 310 ± 5 s^–1^, and *p_I_* = 1.6 ± 0.01%. Notably, these fits resulted in very high ΔR_2,2*st*_ values (>50 s^–1^, Figure 2D) for many residues. Here ΔR_2,2*st*_ is calculated as *R*_2*I*_ – *R*_2*F*_. This broadening is also evident directly in the CEST profiles: for example, the minor-state dips of F41 and V42 are substantially broader than that of Y55, which in turn is broader than the dips observed for L53 and R61 (Figure 2B). Such residue-dependent broadening suggested that the fitted *R*_2*I*_ values may not reflect purely intrinsic relaxation of the intermediate state, but rather an additional exchange contribution arising from unresolved exchange involving the minor state.^17,24,29,30^ Moreover, ^15^N CEST data recorded at 600 and 1200 MHz showed similar broadening of the minor-state dip for several residues (Figure 2D) and the magnitude of ΔR_2,2*st*_ scaled with *B*_0_ field strength (Figure 2D, E), as expected for exchange broadening (eq 3).

### Multi-Field ^15^N CEST Resolves Three State Folding of Im7 under Native Conditions

We next fitted the ^15^N CEST data to a three-state exchange model in which the native state F exchanges with the intermediate state I, and state I undergoes additional exchange with another sparsely populated state, denoted as M. Among the 19 residues used for the initial two-state analysis, eight residues, set8 (E23, H40, F41, V42, I54, Y55, Y56, and D62) showed a monotonic dependence of the apparent two-state chemical shift difference, Δϖ_2*st*_, on *B*_0_ field strength (Figure 2F). The *B*_0_-dependent displacement provides information on the relative chemical shift positions of states I and M, and this set of 8 residues was therefore selected for the three-state analysis. As established by the simulations (Figure 1), the *apparent* I-state dip moves in the direction of the *true* I-state chemical shift with increasing field. For each residue, the sign of Δϖ_*IM*_ = ϖ*_M_* – ϖ*_I_* could therefore be determined from the field dependence of Δϖ_2*st*_. (Figure 2F). For residues E23, H40, F41, and V42, *Δ*ϖ_*IM*_ was positive and for residues I54, Y55, Y56, and D62, *Δ*ϖ_*IM*_ was negative. This sign information was then used in the three-state analysis of the ^15^N CEST data from three fields, 600 MHz *(B*_1_= 16.0 Hz, and 32.0 Hz), 800 MHz (*B*_1_= 23.4, 58.6, 146.5, and 234.5 Hz) and 1200 MHz (*B*_1_= 21.8 Hz, and 54.5 Hz). A triangular model where all three-states, F, I and M can exchange with one another subject to the constraint *R*_2*M*_ = *R*_2_ *_I_* = *R*_2*F*_ reproduced the data well ( $\chi_{red}^{2}=1.17$ ; Figure 3A) with *k*_*ex*,*FI*_ = 430 ± 20 s^–1^, *k*_*ex*,*FM*_ = 50 ± 55 s^–1^, *k*_*ex*,*IM*_ = 5400 ± 450 s^–1^ and *p_I_* = 1.1 ± 0.02%, *p_M_* = 0.37 ± 0.03%. Bootstrap analysis defined all parameters except the direct F↔M exchange rate, which remained statistically indistinguishable from zero (Figure S3).

**Figure 3:**
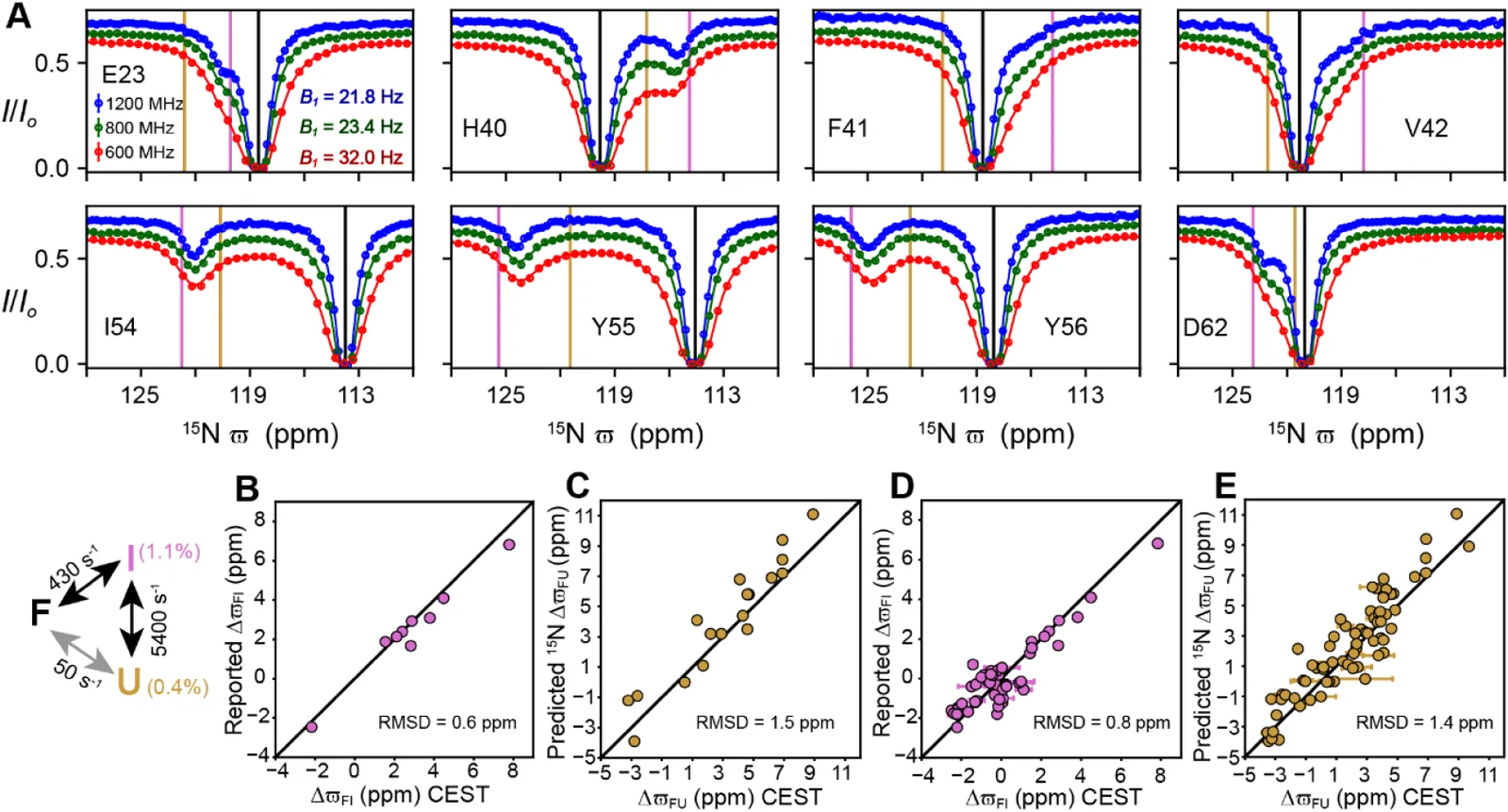
Multi-field ^15^N CEST resolves three-state exchange. A) Three-state fits of the multi-field ^15^N CEST data for eight representative residues. The circles are experimental data points and the connecting lines are the best fit to the three-state exchange model shown in the bottom left. The vertical lines are drawn at the resonance positions of states F (black), I (magenta) and U (gold). B) *Δ*ϖ_*FI*_ obtained from the three-state fits of the CEST data from set18 residues compared with the reported *Δ*ϖ_*FI*_.^44^ Reported *Δ*ϖ_*FI*_ values were calculated as described in the legend of Figure 2. C) Δϖ_*FU*_ obtained from the three-state fits of the CEST data from set18 residues compared with the predicted Δϖ_*FU*_ of WT Im7. The predicted Δϖ_*FU*_ values were calculated as ϖ_*U*,*predicted*_ – ϖ_*F*,*exp*_. Here, ϖ_*U*,*predicted*_ denotes the random-coil ^15^N chemical shifts of WT Im7 predicted using POTENCI,^63^ and ϖ_*F*,*exp*_ denotes the experimental ground-state chemical shifts of WT Im7 measured in the present study. D, E) Same as (B, C), but for all residues 11 to 87.

As an independent test of the sign assignments obtained from the *B*_0_-dependence of Δϖ_2*st*_, we recorded additional ^15^N CEST data at 15 °C and 20 °C at 800 MHz. Previous studies have shown that, in systems with rapid exchange between the two minor states, changing temperature can shift Δϖ_2*st*_ toward one of the minor states and provide information on the relative sign of *Δ*ϖ_*IM*_.^29,30^ Consistent with this expectation, the temperature dependence of Δϖ_2*st*_ for the set8 residues agreed with the sign information obtained from the field dependence of Δϖ_2*st*_ (Figure 2F and Figure S4). Specifically, residues E23, H40, F41, and V42 showed a monotonic increase in Δϖ_2*st*_ with increasing temperature, whereas residues I54, Y55, Y56, and D62 showed a monotonic decrease (Figure S4). Thus, the temperature-dependent CEST data provide an independent check of the *B*_0_-based sign assignment and support the robustness of the proposed multi-field CEST approach.

After establishing a well-defined global three-state exchange model from the set8 residues, we expanded the data analysis to the remaining 11 residues of the set19. To this end, we followed a previously established bootstrapping protocol,^29^ where for each of 200 bootstrap trials, the global parameters *p_I_*, *p_M_*, *k*_*ex,FI*_, *k*_*ex,FM*_, and *k*_*ex,IM*_ were fixed to the values obtained from the set8 analysis (Figure S3), whereas the residue-specific parameters *Δ*ϖ_*FI*_, *Δ*ϖ_*IM*_, and *R*_2*F*_ were fitted independently for each residue. For these 11 residues, the apparent minor state dip did not show a monotonic *B*_0_-dependent displacement of Δϖ_2*st*_ and the sign of *Δ*ϖ_*IM*_ could therefore not be assigned directly from the two-state analysis. Therefore, for each bootstrap trial and each residue, two independent fits were performed: one in which *Δ*ϖ_*IM*_ was constrained to be positive and one in which *Δ*ϖ_*IM*_ was constrained to be negative. The solution with the lower reduced $\chi_{red}^{2}$ value was selected for that trial. For 10 of the 11 residues in this set, one sign of *Δ*ϖ_*IM*_ was selected in more than 80% of the bootstrap trials, indicating a robust preference for one of the two possible solutions (Figure S5). Thus, unambiguous *Δ*ϖ_*FI*_ and *Δ*ϖ_*IM*_ values were obtained for 18 of the 19 residues in the initial set, enabling residue-specific characterization of the folded, intermediate, and newly detected M-state chemical shifts. Constraining the signs of *Δ*ϖ_*IM*_ for these 18 residues (referred to as set18) resulted in an excellent fit to the triangular three-state exchange model $\left(\chi_{red}^{2}=1.1\right)$ .

We next examined the structural nature of the two minor states via an analysis of their chemical shifts. In particular, the chemical shift analysis allowed us to identify the nature of the newly detected M state. The I-state chemical shifts agreed closely with those reported for the Im7 folding intermediate (Figure 3B), confirming the assignment of state I. Note that the I-state chemical shifts determined here from the three-state CEST analysis are not identical to those reported for the trapped-intermediate variant L53A/I54A-Im7.^44^ In the L53A/I54A variant, much of the sequence is subject to chemical exchange on the microsecond–millisecond time scale arising from interconversion between different conformations, which broadens many resonances and precluded assignment of the C-terminal region of helix II and the following loop.^44^ The reported shifts are therefore averaged over interconverting conformations, and are available for only a subset of the residues analyzed here, whereas the values determined here follow directly from the three-state exchange model. The three-state model also explains why the apparent minor-state dip positions obtained from the initial two-state analysis were close to the chemical shifts of the folding intermediate (Figure 2C). State I is substantially more populated than state M (∼1.1%, ∼0.4%); consequently, the apparent minor-state dip lies close to the I-state chemical shift and is displaced only slightly toward the M-state chemical shift by the fast I↔M exchange. The assigned I-state chemical shifts also provide a reference for evaluating the structural nature of state M. The chemical shifts of the M state showed close agreement with predicted random-coil chemical shifts (Figure 3C), identifying it as the unfolded state that is populated at ∼0.4% in fast exchange with the intermediate state I. This remarkable result shows that CEST can provide residue-specific structural information for a state that is not observed as a separate CEST dip, allowing an otherwise hidden component of the folding landscape to be identified directly under native equilibrium conditions.

Having identified that the newly detected state M is the unfolded state of Im7, we extended the analysis to all residues for which ^15^N CEST data were available. The global exchange parameters remained fixed to those obtained from the analysis of set8 residues, whereas residue-specific parameters, such as *R*_1*F*_, *R*_2*F*_, Δϖ_*FI*_, and Δϖ_*FU*_, were allowed to vary. The relaxation rates of the excited states were constrained as *R*_2_ *_I_* = *R*_2*F*_ and *R*_2*U*_ = *R*_2*F*_/2, consistent with the expectation that the intrinsic *R*_2_ of the unfolded state (*R*_2*U*_) should be approximately *R*_2*F*_/2.^24,62^ For residues outside the 18-residue set, the U-state chemical shifts were initialized using predicted random-coil chemical shifts for Im7.^63^ This global fitting procedure resulted in excellent fits for all residues $\left(\chi_{red}^{2}=1.05\right)$ . Across the sequence, the fitted I-state chemical shifts agreed closely with the reported chemical shifts of the Im7 folding intermediate (RMSD = 0.8 ppm, Figure 3D), whereas the fitted U-state chemical shifts were close to predicted random-coil values (RMSD = 1.4 ppm, Figure 3E). The fitted U-state chemical shifts also agreed with previously reported chemical shifts for unfolded-state mimics of Im7, providing an independent benchmark because random-coil values were used to initialize the fits (Figure S6A,B).

### Multi-Field CEST Resolves Exchange That Remains Hidden in CPMG Profiles

Finally, we asked whether CEST was required to resolve the three states of Im7, or whether they could instead be detected by ^15^N CPMG relaxation-dispersion experiments. Three-state exchange processes have previously been characterized using ^15^N CPMG relaxation-dispersion experiments, often in combination with additional experiments.^9,31,32,64–66^ However, when a major state A exchanges with a minor state B that undergoes rapid exchange with a more sparsely populated state C, the C-state contribution may become effectively hidden in conventional ^15^N CPMG profiles, yielding an apparent A↔B exchange process.^29,30,32^ To test this scenario for Im7, we simulated ^15^N TROSY-CPMG^16,67–69^ profiles at 600 and 800 MHz using the exchange parameters and residue-specific *Δ*ϖ_*FI*_ and Δϖ_*FU*_ values obtained from the three-state CEST analysis (see Supplementary Information for details). Indeed, the simulated profiles were well described by an apparent F↔I model, yielding *k_ex,FI_* = 455 ± 10 s^–1^, *p_I_* = 1.1 ± 0.02%, and $\chi_{red}^{2}=1.1$ (Figure 4A, B). The apparent I-state chemical shifts obtained from these fits agreed well with the I-state chemical shifts from the three-state CEST analysis (Figure 4C) and with previously reported Im7 intermediate-state shifts (Figure 4D).

**Figure 4:**
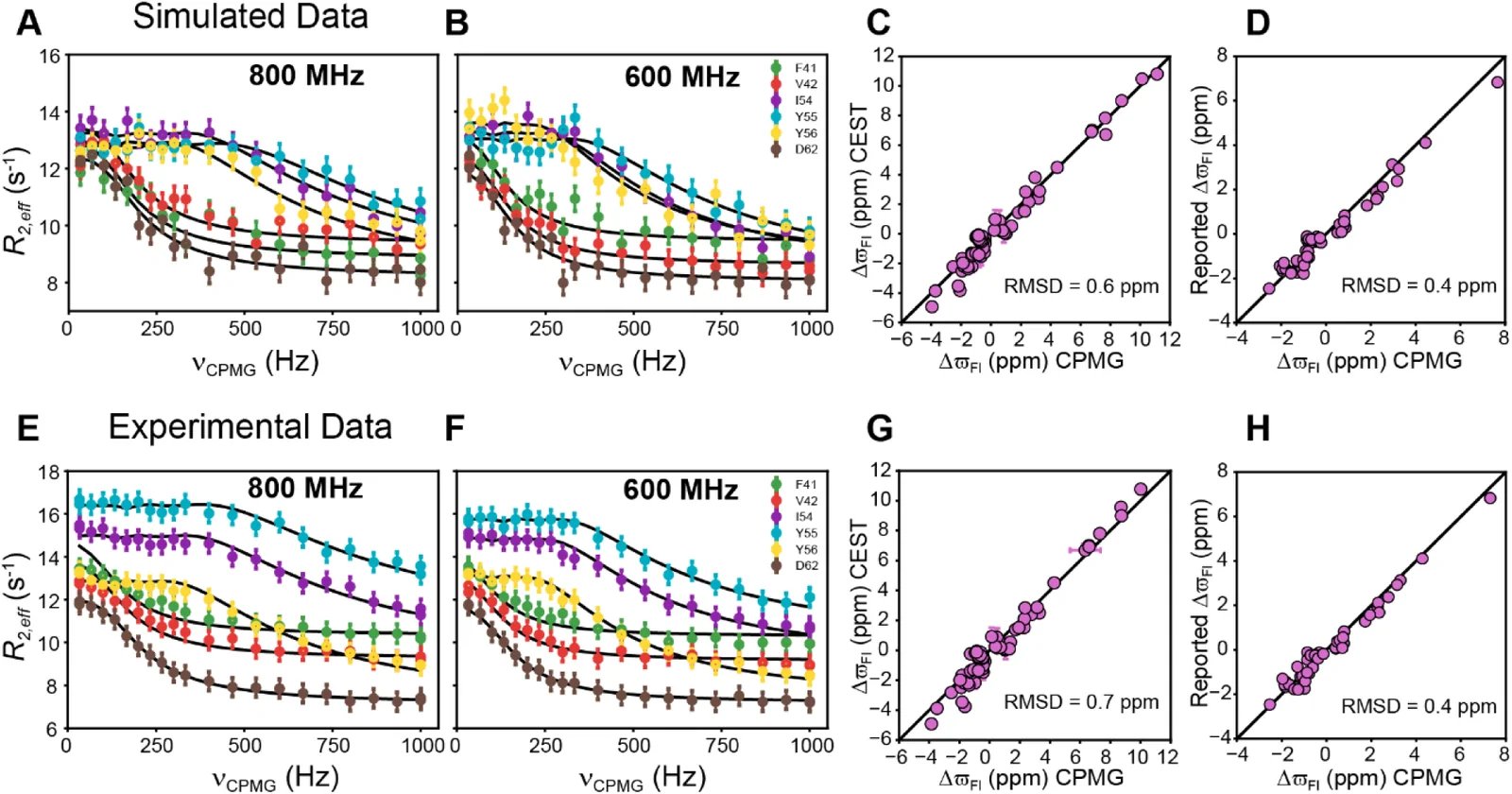
Inability of ^15^N TROSY CPMG to resolve the three-state exchange. A, B) Simulated ^15^N TROSY-CPMG relaxation-dispersion profiles at 800 MHz (A) and 600 MHz (B) for six representative residues. Profiles were simulated using the three-state exchange parameters obtained from the CEST analysis, Gaussian noise was added to the data such that the minimum uncertainty in *R*_2,*eff*_ was 1.5% of *R*_2,*eff*_ or 0.3 s^–1^, whichever was larger, and then data was fitted with an apparent two-state F↔I exchange model. Simulated data points are shown as coloured circles while solid lines are calculated from best fit parameters, *k*_*ex,FI*_ = 455 ± 10 s^–1^, *p_I_* = 1.1 ± 0.02%. C, D) Comparison of *Δ*ϖ_*FI*_ values obtained from the two-state fits of the simulated CPMG profiles with the corresponding *Δ*ϖ_*FI*_ values from the three-state CEST data analysis (C) and previously reported Im7 intermediate-state chemical shift differences (D). E–H, Same as (A–D), but for experimental ^15^N TROSY-CPMG relaxation-dispersion profiles of WT Im7 recorded at 10 °C. In E and F, experimental data points are shown as coloured circles while solid lines are calculated from best fit parameters, *k*_*ex,FI*_ = 475 ± 10 s^–1^, *p_I_* = 1.2 ± 0.02%.

We then tested this prediction experimentally by recording ^15^N TROSY-CPMG^68,69^ profiles of WT Im7 at 600 and 800 MHz at 10 °C. Consistent with the simulations, the experimental CPMG profiles were well described by an apparent F↔I model yielding exchange parameters (*k*_*ex*,*FI*_ = 475 ± 10 s^–1^, *p_I_* = 1.2 ± 0.02%, and $\chi_{red}^{2}=0.5$ ; Figure 4E, F), and the fitted I-state chemical shifts again agreed with those obtained from the three-state CEST analysis (Figure 4G) and with previously reported intermediate-state chemical shifts (Figure 4H). Because the two-state model reproduced the CPMG profiles within the expected uncertainty and no systematic deviations remained, the CPMG data alone provide no basis for introducing a third state. These results explain why previous CPMG studies^45,47,70^ of Im7 could be described by a simple F↔I exchange scheme. In contrast, CEST remains sensitive to the additional I↔U exchange through the position and line width of the minor-state dip, highlighting its particular utility for detecting fast exchange between sparsely populated states. A detailed comparison of the detectability of state U by CEST and CPMG is provided in the Supporting Information (see section “Detectability of minor exchange by ^15^N CEST versus ^15^N CPMG” and Figure S7).

## Discussion

Multi-field CEST introduced in this work resolves a central ambiguity in the analysis of fast exchange between sparsely populated states. Exchange broadening of a minor state dip identifies additional dynamics, and the *B*_0_-dependence of the dip supplies the sign information needed to distinguish otherwise degenerate three-state solutions. For Im7, multi-field CEST thus made it possible to directly characterize an F↔I↔U exchange process under equilibrium conditions: the folded state exchanges with a ∼1% folding intermediate, which exchanges rapidly with a ∼0.4% unfolded state. The approach thereby recovers the population, kinetics, and residue-specific chemical shifts of a state that does not appear as a separate CEST dip.

The residue-specific ^15^N chemical shifts provide a direct structural description of the Im7 intermediate at equilibrium (Figure 5A, B). Large folded-to-intermediate chemical shift differences cluster in helix H3 and at the C-terminal end of H2, whereas intermediate-to-unfolded differences extend across most of the sequence but remain small in H3. Thus, helices H1, H2, and H4 are substantially structured in the intermediate state, whereas H3 remains largely disordered. This residue-level picture is fully consistent with ϕ-value analysis, equilibrium hydrogen-exchange and NMR studies of the trapped intermediate L53A/I54A.^43,46,59,61^ The agreement is important because the present chemical shifts are obtained in a perturbation-free manner for wild-type Im7 under native equilibrium conditions rather than from a stabilized intermediate mimic.

**Figure 5:**
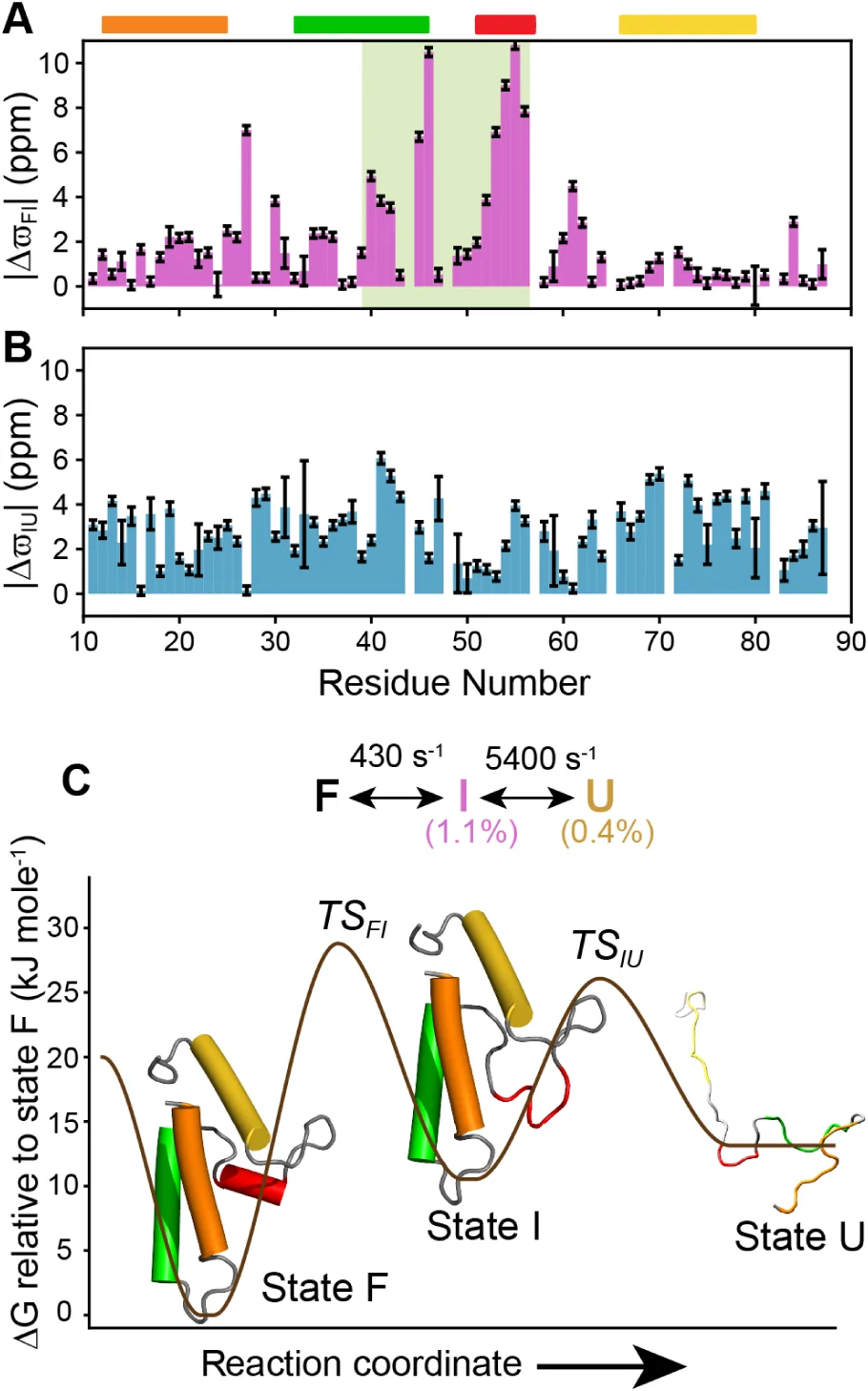
Reconstruction of the Im7 folding energy landscape from multi-field CEST. A) Absolute chemical shift differences between the folded and intermediate states, |Δϖ_*FI*_|, for residues 11–87. Residues with |Δϖ_*FI*_| > 3 ppm are primarily located in the H3 region and C-terminal of H2. B) Absolute chemical shift differences between the intermediate and unfolded states, |Δϖ_*IU*_| for residues 11–87. C) Free-energy landscape of Im7 folding constructed from the parameters obtained from the CEST data analysis. Structural models are based on the experimental structure of Im7 [PDB: 1AYI] and the chemical shifts determined in A and B.

The equilibrium populations and microscopic rate constants define the equilibrium free-energy landscape of Im7 (Figure 5C). The determined Δ*G*_*FI*_ = 10.5 ± 0.1 kJ mol^–1^ and Δ*G*_*FU*_ = 13.2 ± 0.2 kJ mol^–1^ agree well with values derived from kinetic folding measurements at pH 7.0 and 10 °C, which were reported as Δ*G*_*FI*_ = 10.9 ± 1.7 kJ mol^–1^ and Δ*G*_*FU*_ = 16.3 ± 1.3 kJ mol^–1^.^71^ Not only the thermodynamic parameters, but also the kinetic parameters agree with previously reported findings. The I→F rate, *k*_*IF*_ = 425 ± 20 s^–1^, is in close agreement with the value of ∼300 s^–1^ reported from ultrarapid-mixing experiments.^57^ Similarly, the U→I rate, *k*_*UI*_ = 4060 ± 400 s^–1^, matches the previously reported value of ∼3000 s^–1^.^57^ This agreement is particularly notable because the two approaches probe the folding landscape in fundamentally different ways. The kinetic experiments follow the relaxation of Im7 after perturbation of the folding equilibrium, whereas multi-field CEST detects the exchange between states at equilibrium. Although all pairwise transitions were allowed during the fit, the direct F↔U exchange rate was indistinguishable from zero within experimental uncertainty, supporting an effectively sequential pathway. Notably, this topology is consistent with ultrarapid-mixing studies and molecular dynamics simulations of Im7 folding, in which the intermediate was found to be the dominant folding route.^57,60^ The key achievement of the NMR CEST analysis is that all these conclusions come from a single equilibrium experiment and that they are resolved to the single residue level.

The landscape also separates structural progress from thermodynamic stabilization. The intermediate is native-like across much of the sequence, particularly in helices H1, H2, and H4, yet lies only ∼3.0 kJ mol^–1^ below the unfolded state, but ∼10.0 kJ mol^–1^ above the folded state (Figure 5C). Formation of substantial amounts of secondary structure in the U→I transition is therefore minimally exergonic and thermodynamic stabilization of the fold occurs mainly during formation of the full three-dimensional structure in the I→F transition. Similar decoupling of structural progress and thermodynamic stabilization has been observed for Fyn SH3 and the HYPA/FBP11 FF domain, where partially structured intermediates precede the major decrease in free energy associated with the native state consolidation.^9,24,28,72^

Also in terms of kinetics, the Im7 folding landscape fits into a broader picture emerging from study of the small four-helix-bundle FF domain, which collapses from the unfolded state on the ∼400 μs time scale to a folding intermediate in which three out of four helices are formed.^24,28,38,72^ This intermediate then converts to the native state by a slower transition on the millisecond time scale.^28,38,73^ Plausibly, the partially structured intermediate formed during the rapid phase of Im7 folding remains loosely packed and is stabilized by a combination of native and non-native interactions.^43,44,57^ Reorganization in the final folding step and consolidation of the native tertiary core requires the specific reordering of the native versus the non-native contacts, which takes longer but also delivers larger thermodynamic stabilization.

In summary, multi-field CEST employs the magnetic-field dependence of the apparent minor-state dip position in CEST profiles to remove a substantial ambiguity of three-state models in a perturbation-free manner. This approach is conceptually related to previous strategies that use field-dependent exchange-induced peak shifts to resolve chemical-shift sign ambiguities in relaxation-dispersion experiments.^53,74^ Multi-field CEST converts the broadening of a minor-state dip from a qualitative indicator of hidden exchange into a quantitative route for obtaining populations, exchange rates, and state-specific chemical shifts. Multi-field CEST will be broadly useful for studying fast exchange processes between sparsely populated states. The method proposed here is not restricted to ^15^N nuclei, but can be readily applied to other spin probes, including methyl ^13^C, and to biomolecules beyond proteins, such as RNA and DNA.

## Supplementary Material



## Data Availability

The processed CEST and CPMG data, and the *ChemEx* input files used in this study are openly available on Zenodo at 10.5281/zenodo.22794667.

## Notes

Detailed procedures for protein expression and purification, NMR experiments and data analysis, supplementary figures and tables, and residue-specific ^15^N chemical shifts of the folded, intermediate, and unfolded states (PDF)
