# Supplementary material for: Multi-Field CEST NMR Reconstructs a Protein Folding Landscape
Source: J Am Chem Soc. 2026 Oct 2;148(40):43873–82. doi: 10.1021/jacs.6c16569 (PMC13674274; doi:10.1021/jacs.6c16569)
Supplement: Supplementary file 1 [file ja6c16569_si_001.pdf]

Supporting Information

Multi-Field CEST NMR Reconstructs a Protein Folding Landscape

Ved Prakash Tiwari[1*], Laetitia Rožić[1], Sebastian Hiller[1*]

[1]Biozentrum, University of Basel, Spitalstrasse 41, 4056 Basel, Switzerland

This file contains:

- Materials and Methods
- Figures S1 – S7
- Tables S1 – S2
- Supplementary References

Materials and Methods

Protein Expression and Purification

Im7 was expressed and purified as described previously,[1] with minor modifications. Briefly, uniformly ^{15}N-labeled WT Im7 was expressed in *E. coli* BL21(DE3) Lemo cells from a His$_9$-TEV-Im7 construct. Cells were grown in M9 minimal medium supplemented with 1 g/L $^{15}NH_4Cl$ as the sole nitrogen source and 4 g/L unlabelled glucose as the sole carbon source. Protein expression was induced with 0.5 mM IPTG at an OD_{600} of approximately 0.9 and continued for 15 h at 25 °C. Cells were harvested by centrifugation, resuspended in lysis buffer containing 25 mM HEPES, pH 7.0, and 150 mM NaCl, and lysed by French press. The lysate was cleared by centrifugation. His$_9$-TEV-Im7 was purified by Ni-IMAC, washed with lysis buffer containing low imidazole, and eluted with 500 mM imidazole. The eluted protein was dialyzed against lysis buffer and incubated with TEV protease in the presence of 0.5 mM EDTA and 1 mM DTT to remove the His$_9$ tag. Cleaved Im7 was separated from His-tagged material by reverse Ni-IMAC and further purified by size-exclusion chromatography in 20 mM sodium phosphate, pH 7.0, and 50 mM NaCl. Fractions containing pure Im7 were pooled, concentrated to 750 µL at approximately 1 mM, and buffer-exchanged into NMR buffer containing 50 mM potassium phosphate, pH 6.6, and 0.02% (w/w) sodium azide. To minimize complications arising from H/D exchange in the CEST profiles,[2] 2% (v/v) D_2O was used for the spectrometer lock. The final sample was divided into two aliquots: 550 µL was transferred to a 5 mm NMR tube, and 200 µL was transferred to a 3 mm NMR tube.

NMR spectroscopy

Except for the temperature-dependent CEST experiments (Table S1), all NMR experiments were carried out at 10 °C (unless stated otherwise) on Bruker 600, 800, and 1200 MHz spectrometers equipped with cryogenically cooled triple-resonance probes with Z-axis gradient. Experiments at 600 and 800 MHz were recorded using 5 mm probes, whereas experiments at 1200 MHz were recorded using a 3 mm probe. ^{15}N CEST experiments were acquired using the previously described pulse sequence of Vallurupalli et al. (JACS, 2012),[3] with $90_x240_y90_x$ composite ^{1}H decoupling applied during the T_{ex} period. For each dataset, a reference plane with T_{ex} = 0 was recorded to obtain reference peak intensities (I_0). The strengths of the B_1 fields applied during the T_{ex} periods were calibrated using the nutation method of Guenneugues et al. (JMR, 1999).[4] Additional experimental details are provided in

Table S1. ^{15}N TROSY-CPMG experiments were recorded at 600 and 800 MHz using the [0013] phase-cycled implementation.[5–8] A constant relaxation period of T_{ex}= 30 ms was used, and the CPMG pulse frequency, (ν_{CPMG}), was varied from 33 to 1000 Hz. A total of 23 planes were recorded for each dataset, including two duplicate planes for error estimation and a reference plane recorded without the CPMG relaxation period to obtain reference peak intensities. The indirect ^{15}N dimension was acquired with a spectral width of 18 ppm and 42 and 48 complex points at 600 and 800 MHz, respectively. The actual sample temperature inside the probe was calibrated independently by inserting an NMR tube containing Milli-Q water and a thermocouple into the probe. In addition, RF-induced sample heating during ^{1}H decoupling was evaluated directly, since ^{1}H decoupling during the T_{ex} period of ^{15}N CEST experiments can lead to measurable sample heating.[9–11] Because different ^{1}H decoupling field strengths were used at different static magnetic fields, the effective sample temperature was calibrated separately for each experimental condition. Heating was estimated from one-dimensional ^{1}H spectra using the temperature-dependent chemical shifts of well-resolved methyl ^{1}H resonances as internal probes, as described previously for relaxation-dispersion measurements.[9,12] Briefly, a reference 1D ^{1}H spectrum was recorded at the nominal experimental temperature. The CEST experiment was then run under the same RF duty cycle used for data acquisition until steady-state heating was reached, after which a second 1D ^{1}H spectrum was recorded immediately. A third 1D ^{1}H spectrum was acquired after increasing the probe temperature by 1 °C. The displacement of selected CH_3 ^{1}H resonances after the RF-heating period was compared with the corresponding displacement produced by the 1 °C temperature increase. The RF-induced temperature change was calculated from the ratio of these chemical-shift differences and used to adjust the nominal spectrometer temperature such that the effective sample temperature was matched between datasets recorded at different magnetic fields and decoupling powers. Since no ^{1}H decoupling was applied during the relaxation period of the ^{15}N TROSY-CPMG experiments, the nominal temperature for these experiments was adjusted such that the actual sample temperature matched that used for the corresponding CEST datasets.

Data analysis

All NMR data were processed with NMRPipe.[13] Spectra were visualized and peaks were labelled using NMRFAM-Sparky.[14] Peak intensities were extracted with PINT, either as a function of the B_1 offset for CEST experiments or as a function of ν_{CPMG} for TROSY-CPMG

experiments.[15] For the TROSY-CPMG data, effective transverse relaxation rates, R_{2eff}, were calculated from the intensities as $\frac{-1}{T_{ex}}\ln\left(\frac{I(\nu_{CPMG})}{I_0}\right)$, where T_{ex} is the constant-time relaxation period, $I(\nu_{CPMG})$ is the peak intensity at a given CPMG frequency, and I_0 is the intensity in the reference spectrum recorded with T_{ex} = 0. Uncertainties in the peak intensities were estimated from duplicate measurements. A minimum uncertainty corresponding to 0.3 s^{-1} or 1.5% of R_{2eff}, whichever was larger, was used for peak intensities. For the CEST data, uncertainties in peak intensities were estimated from the flat regions of the CEST profiles.

All CEST and TROSY-CPMG data were analysed using *ChemEx* (https://github.com/gbouvignies/chemex),[16] which numerically integrates the Bloch–McConnell equations for a specified exchange model and optimizes the model parameters by minimizing $\sum_{i=1}^{N}\left(\frac{I_{calc,i}-I_{exp,i}}{\sigma_i}\right)^2$. Here, $I_{exp,i}$ and σ_i are the experimental intensity and its uncertainty for data point i, respectively, and $I_{calc,i}$ is the corresponding intensity calculated from the fitted exchange model. The summation extends over all experimental data points included in the fit. As described in the main text, ^{15}N CEST data recorded at each magnetic field were first analysed separately using a two-state exchange model. For the two-state fits at 600 and 800 MHz, 19 residues with $|\Delta\varpi_{2st}| > 2$ ppm were included: E21, E23, V27, T30, D35, H40, F41, V42, T45, E46, D52, L53, I54, Y55, Y56, N60, R61, D62, and F84. For the 1200 MHz data, the same residue set was used except for V27 and T45, which were excluded because of very low signal-to-noise, resulting in a final set of 17 residues. In these fits, the exchange rate $k_{ex,FI}$ (k_{FI} + k_{IF}) and the minor-state population p_I were treated as global parameters shared by all residues fitted together. The chemical shifts of the major and minor states, together with the relaxation rates R_{1F}, R_{2F}, and, in some cases, R_{2I}, were treated as residue-specific parameters. R_{1I} was constrained to be equal to R_{1F} and does not affect the analysis.[3] While analysing the ^{15}N CEST data recorded at temperatures 15 °C and 20 °C (800 MHz), the same set of 19 residues were used for the two-state fits of the data. Uncertainties in the two-state best-fit parameters were estimated from 100 Monte Carlo trials or from the covariance matrix.

For the three-state analysis, ^{15}N CEST data recorded at 600, 800, and 1200 MHz were fitted together. As described in the text, the initial three-state fits were performed using residues that showed a monotonic change in the apparent two-state chemical-shift difference, $\Delta\varpi_{2st}$, with B_0 field strength: E23, H40, F41, V42, I54, Y55, Y56, and D62. The exchange rates $k_{ex,FI}$,

$k_{ex,FM}$, and $k_{ex,IM}$, together with the populations p_I and p_M, were treated as global parameters. The state-specific chemical shifts ϖ_F, ϖ_I, and ϖ_M, and the ground-state relaxation rates R_{1F} and R_{2F}, were treated as residue-specific parameters. The relaxation rates of the two minor states were constrained to be equal to the corresponding ground-state relaxation rates. Chemical shifts were treated as field independent, whereas relaxation rates were allowed to vary with magnetic field. During three-state fitting, $\Delta\varpi_{FI}$ and $\Delta\varpi_{IM}$ were constrained to lie within ±20 ppm to avoid unphysical solutions. Initial values for $\Delta\varpi_{FI}$ were taken from the two-state fits of the CEST data. The sign of $\Delta\varpi_{IM}$ was constrained from the B_0 dependence of the apparent two-state minor-state chemical-shift difference, $\Delta\varpi_{2st}$, and $\Delta\varpi_{IM}$ was initialized to either +3 or −3 ppm according to this sign. Uncertainties in the best-fit parameters from the three-state CEST analysis were estimated from 200 bootstrap trials. In each trial, the experimental ^{15}N CEST data were resampled with replacement, while keeping the total number of data points unchanged. Each resampled data set was fitted independently to the same three-state exchange model, using the same parameter constraints and fitting protocol as for the original data. The reported parameter uncertainties were obtained from the distributions of fitted values over the 200 bootstrap trials.

The ^{15}N TROSY-CPMG data recorded at 600 and 800 MHz for residues 11–87 were fitted simultaneously to a two-state F↔I exchange model. The exchange rate, ($k_{ex,FI}$), and the minor-state population, p_I, were treated as global parameters shared by all residues. The ^{15}N chemical-shift of the I state, ϖ_I, and intrinsic transverse relaxation rate, $R_{2F}(\nu_{CPMG} = \infty)$ of the F state were the residue specific parameters. Uncertainties in the best-fit parameters were estimated from 100 Monte Carlo trials as implemented in *ChemEx*.

Random coil chemical shifts of Im7 were predicted using the program POTENCI.[17] In Figure 5 the free energy of state K, where K denotes I or U, relative to the folded state F was calculated from the fitted populations according to $\Delta G_{FK}=-RTln(p_K/p_F)$. p_F and p_K are the equilibrium populations of the native state F and state K, respectively, obtained from the fits of ^{15}N CEST data. The free energy of the transition state separating states K and L, ΔG_{FTSKL}, was calculated from the fitted populations and rate constants according to $\Delta G_{FTSKL} = -RTln(p_K/p_F) - RTln(k_{KL}/A)$, using a pre-exponential factor A = 10^6 s^{-1}, where p_F, p_K are the populations of the native state F and state K, and k_{KL} is the rate constant for K→L transition. R is the gas constant and T is temperature in kelvin. The unfolded-state model of Im7 shown in Figure 5C was generated using the program protSA.[18]

Generation of synthetic ^{15}N CEST data

^{15}N CEST profiles for two- and three-state exchange processes depicted in Figure 1 were generated with *ChemEx* with R_1, R_2 of 1 s^{-1} and 15 s^{-1}, respectively for all the states at all B_0 fields. No noise was added to the data. To make the B_0 vs $\Delta R_{2,2st}$ and B_0 vs $\Delta\varpi_{2st}$ plots the B_1 = 17 Hz and B_1 = 35 Hz data were fitted to a two-state (A↔B) exchange model where $k_{ex,AB}$, p_B, ϖ_A, ϖ_B, R_{1A}, R_{2A} and R_{2B} were allowed to float while R_{1B} was constrained to be equal to R_{1A}. To make Figure S1 synthetic ^{15}N CEST profiles were generated with *ChemEx* for a two-state exchange process, A↔B, using exchange parameters similar to those obtained from two-state fits of the WT Im7 ^{15}N CEST data. The exchange rate and minor-state population were set to $k_{ex,AB}$ = 300 s^{-1} and p_B = 1.6 %, respectively. Data were simulated at 600, 800, and 1200 MHz for 58 sites. To obtain a realistic distribution of chemical-shift differences, site-specific $\Delta\varpi_{AB}$ values were taken from the unfolded state of the A39G FF domain.[19] The chemical shift of state A was set to 0 ppm for all sites. Relaxation rates for state A were set to values similar to those obtained for WT Im7 under the conditions used here. R_1 and R_2 were set to 1.4 and 11 s^{-1} at 600 MHz, 1.0 and 15 s^{-1} at 800 MHz, and 0.7 and 20 s^{-1} at 1200 MHz, respectively, for all sites. R_1 for state B was set equal to R_1 of state A for all sites. To mimic the exchange broadening observed for the apparent minor-state dips in the experimental Im7 CEST profiles, R_2 of state B was set to 100, 150, and 250 s^{-1} at 600, 800, and 1200 MHz, respectively. For each magnetic field, ^{15}N CEST profiles were simulated at two saturation field strengths and exchange periods: B_1 = 17 Hz with T_{ex} = 450 ms, and B_1 = 35 Hz with T_{ex} = 400 ms. For the 17 Hz data, saturation offsets were sampled over approximately ±18 ppm around the carrier, which was placed at 0 ppm, using 25 Hz increments. For the 35 Hz data, the same offset range was sampled using 40 Hz increments. Gaussian random noise was added to the simulated intensities with standard deviations of 0.35%, 0.35%, and 0.5% at 600, 800, and 1200 MHz, respectively, matching the experimental noise levels estimated for the WT Im7 data. The synthetic CEST profiles were fitted with a two-state exchange model. During fitting, R_{1A} was allowed to vary, with R_{1B} constrained to be equal to R_{1A}. The chemical shifts of states A and B were also fitted, and both R_{2A} and R_{2B} were allowed to vary. To estimate the precision of the two-state chemical-shift differences, $\Delta\varpi_{AB}$, obtained from the two-state fits of the ^{15}N CEST data, 100 independent synthetic data sets were generated with different realizations of Gaussian noise and fitted independently. For each site with $|\Delta\varpi_{2st}|$ > 2 ppm, the uncertainty in $\Delta\varpi_{2st}$ was estimated as the standard deviation of the fitted values obtained from these 100 fits.

Generation of synthetic ^{15}N TROSY CPMG data

Synthetic ^{15}N TROSY-CPMG data shown in Figure 4A–B were generated with *ChemEx* at 600 and 800 MHz using the [0013] phase-cycling scheme.[5,6,8] Simulations were performed for the 71 residues included in the CEST data analysis. The exchange parameters, together with the residue-specific $\Delta\varpi_{FI}$ and $\Delta\varpi_{FU}$ values, were taken from the best-fit three-state analysis of the ^{15}N CEST data. Relaxation parameters required for the ^{15}N TROSY-CPMG simulations were estimated using an isotropic rotational correlation time, τ_C, of 8 ns. This value was estimated from the R_1 and R_2 values extracted from the CEST data analysis.[20–22] All experimental parameters, including the ν_{CPMG} values, exchange period T_{ex}, ^{15}N CPMG 90° pulse width, pw_{90}, etc. were kept identical to those used for the experimental ^{15}N TROSY-CPMG measurements described above. To account for experimental noise, Gaussian random errors were added to the calculated intensities such that the corresponding uncertainty in $R_{2,eff}$ was 1.5% of the effective relaxation rate. When 1.5 % of $R_{2,eff}$ was smaller than 0.3 s^{-1}, a minimum uncertainty of 0.3 s^{-1} was used. The resulting synthetic CPMG data were then fitted with the same two-state F↔I exchange model and fitting protocol used for the experimental TROSY-CPMG data.

Supplementary Figures

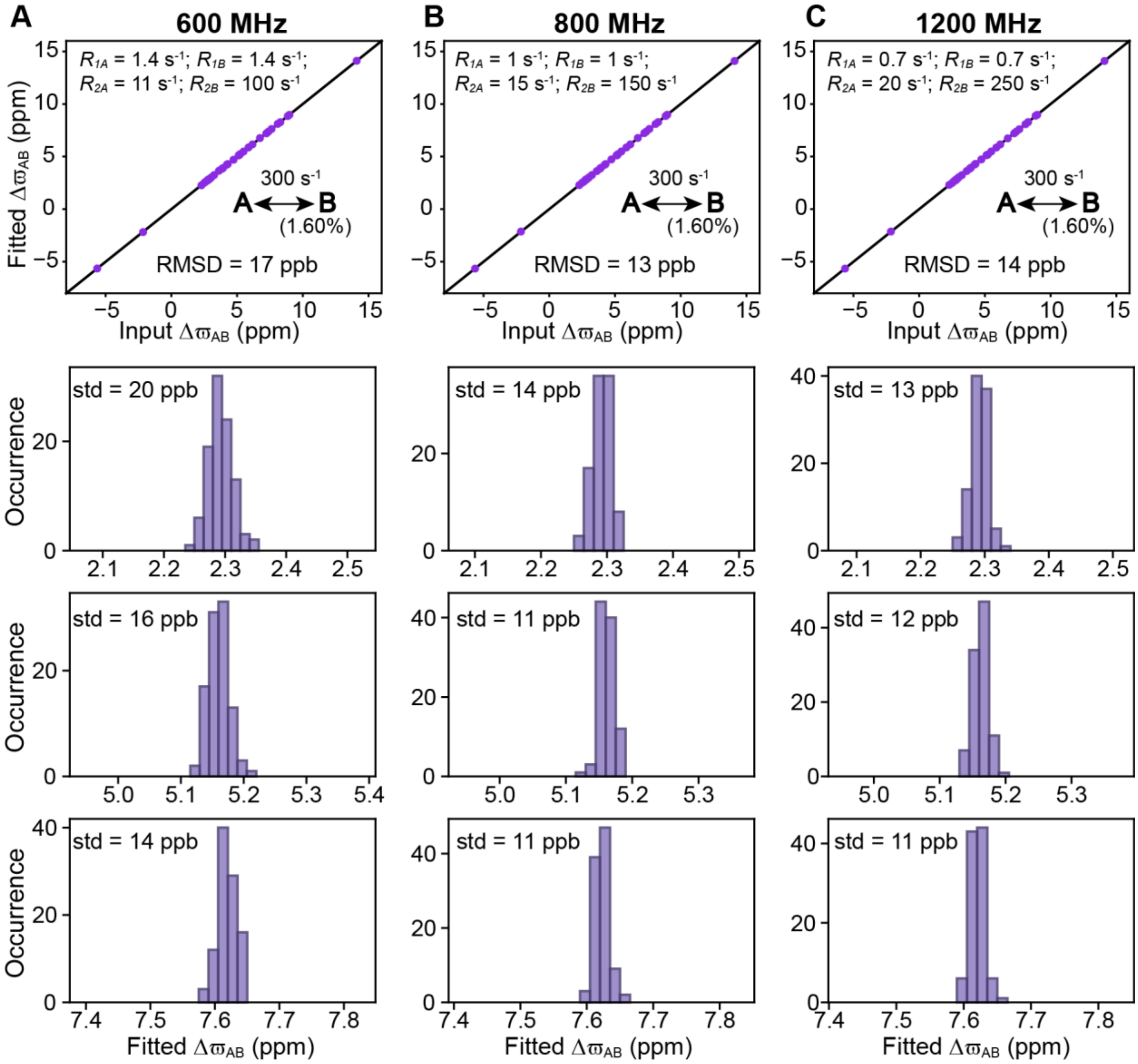

Figure S1. Minor-state dip position in ^{15}N CEST profiles can be determined with high precision. (A–C) Synthetic two-state CEST datasets generated for an A↔B exchange process with k_{ex} = 300 s^{-1} and p_B =1.6 % at proton Larmor frequencies of 600 MHz (A), 800 MHz (B), and 1200 MHz (C). The longitudinal and transverse relaxation rates used at each field are indicated. Top: Chemical-shift differences obtained from two-state fits plotted against their input values. Note that only sites with $|\Delta\varpi_{AB}|$ > 2 ppm were used during the fits. The root-mean-square deviation between fitted and input values is reported for each field. Bottom: Distributions of fitted $\Delta\varpi_{AB}$ values obtained from 100 Monte-Carlo realizations for three representative input chemical-shift differences. The standard deviation of each distribution is indicated, demonstrating that the apparent minor-state chemical-shift position can be determined with high precision at all three magnetic fields.

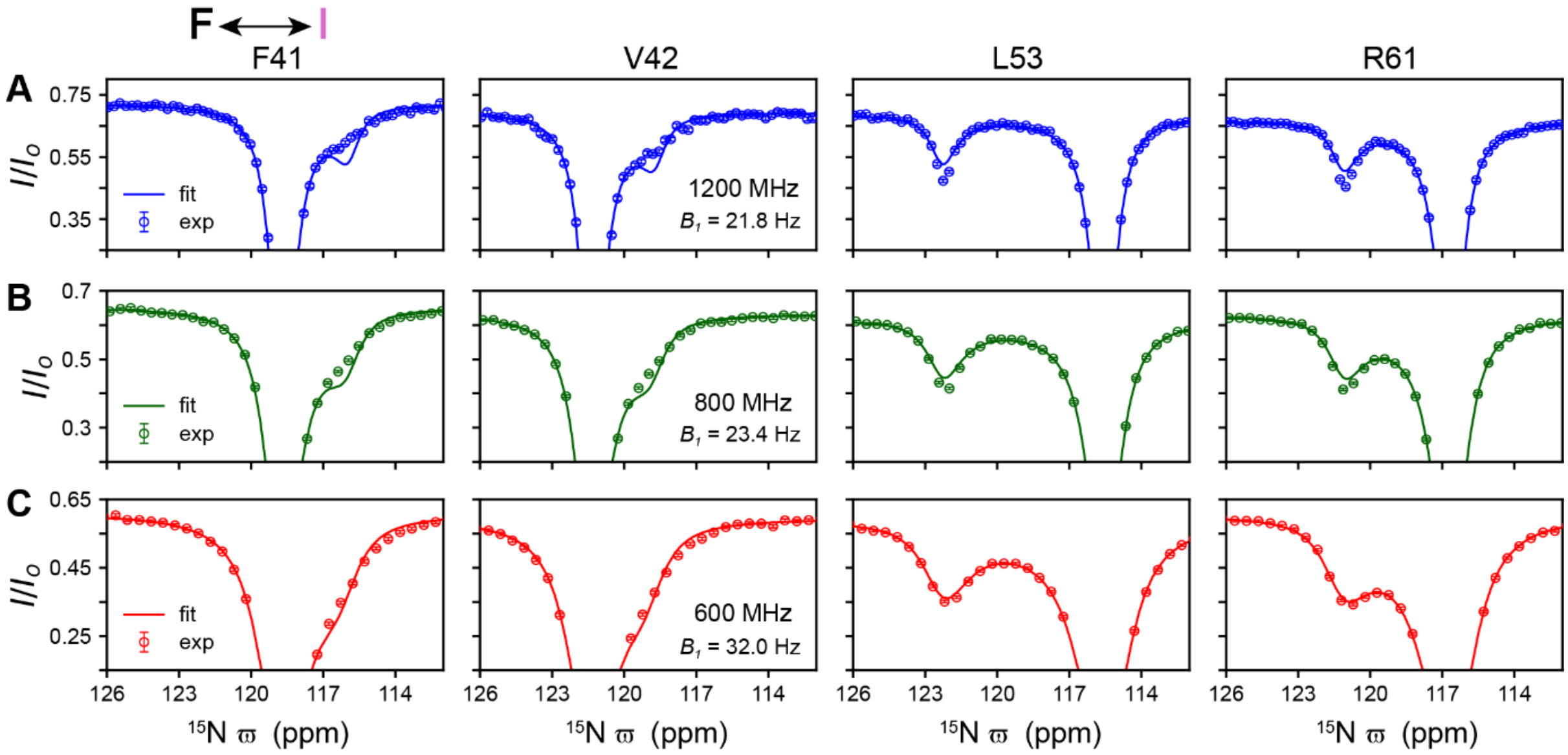

Figure S2. Failure of a simple two-state exchange model to describe the ^{15}N CEST profiles of Im7 WT. (A–C) Representative ^{15}N CEST profiles for residues F41N, V42N, L53N, and R61N, recorded at proton Larmor frequencies of 1200 MHz (A), 800 MHz (B), and 600 MHz (C). The CEST profiles recorded at each B_0 field were analysed independently using a two-state F↔I exchange model, with the transverse relaxation rate of the intermediate state I constrained to be equal to that of the folded state F. Experimental data points are shown as coloured circles with error bars, and solid lines are calculated from the best-fit parameters. Despite the presence of only a single detectable minor-state dip, systematic deviations between the experimental profiles and the two-state fits demonstrate that the exchange process is more complex than simple two-state interconversion between the folded and intermediate states.

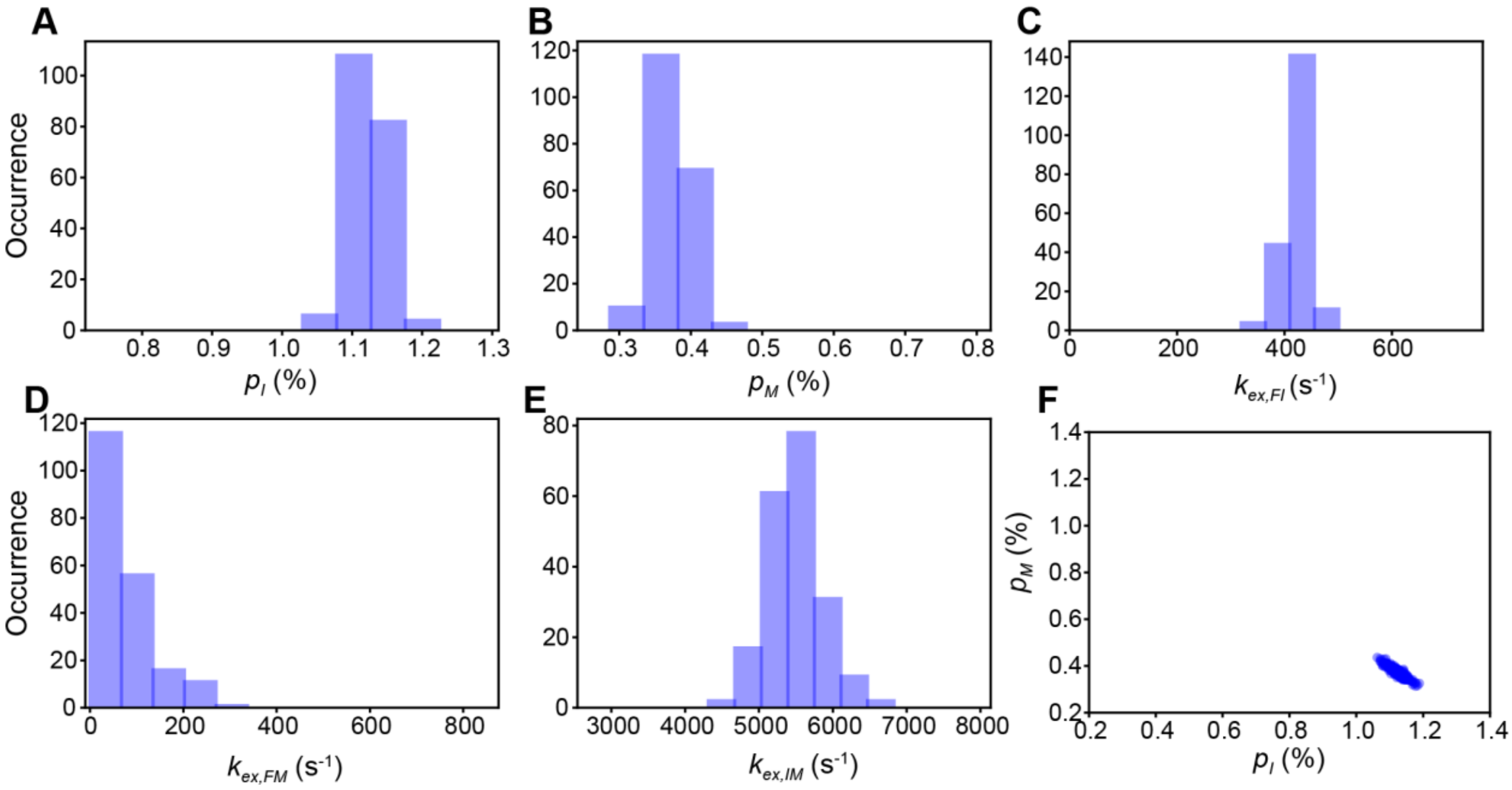

Figure S3. Robustness of the three-state analysis of the ^{15}N CEST data of Im7. (A–F) Distributions of the parameters from 200 bootstrap fits. (A) Population of the intermediate state, p_I; (B) population of the second minor state, p_M; (C) Exchange rate $k_{ex,FI}$, (D) $k_{ex,FM}$, and (E) $k_{ex,IM}$. (F) Joint distribution of p_I and p_M across the 200 bootstrap fits, showing that there is no significant correlation between the fitted populations of the two minor states.

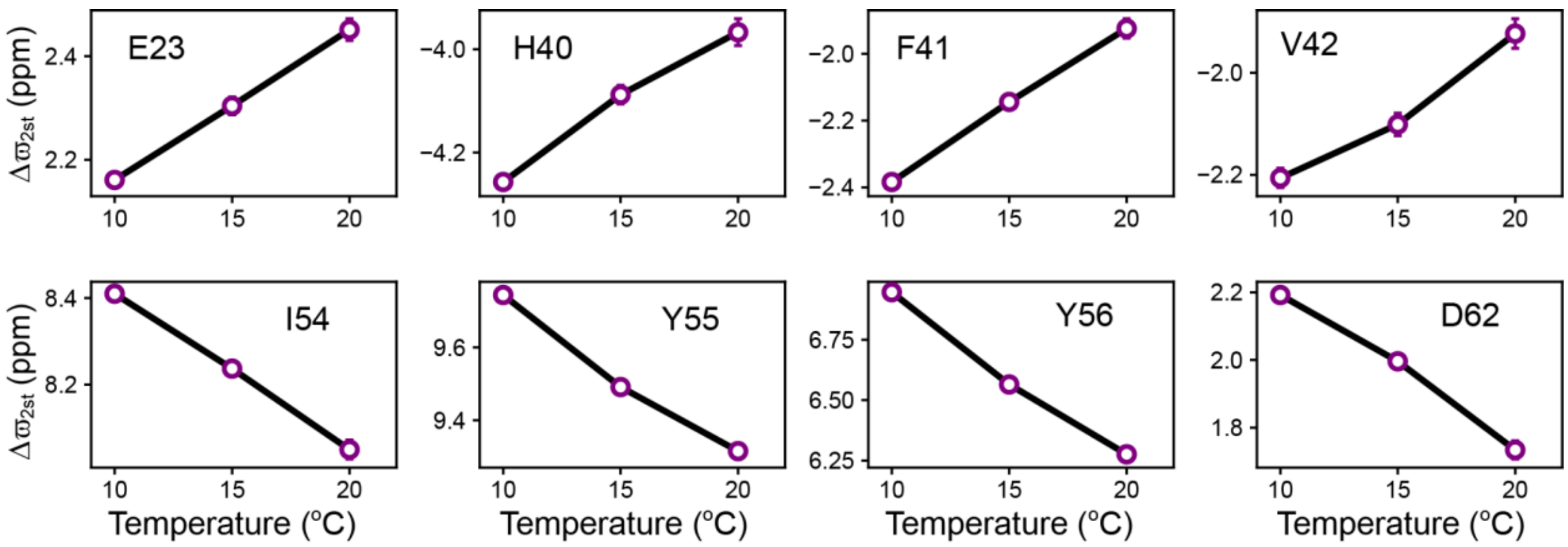

Figure S4. Temperature dependence of the $\Delta\varpi_{2st}$ obtained from two-state analysis of ^{15}N CEST data. ^{15}N CEST profiles recorded at each temperature were fitted independently to a two-state exchange model, yielding the apparent chemical-shift difference, $\Delta\varpi_{2st}$, between the folded and minor states. The temperature dependence of $\Delta\varpi_{2st}$ is shown for residues E23, H40, F41, V42, I54, Y55, Y56, and D62. Purple open circles represent the fitted values, with error bars indicating the corresponding fitting uncertainties, and solid black lines connect the data points to highlight their systematic temperature dependence. Further experimental details are provided in Table S1.

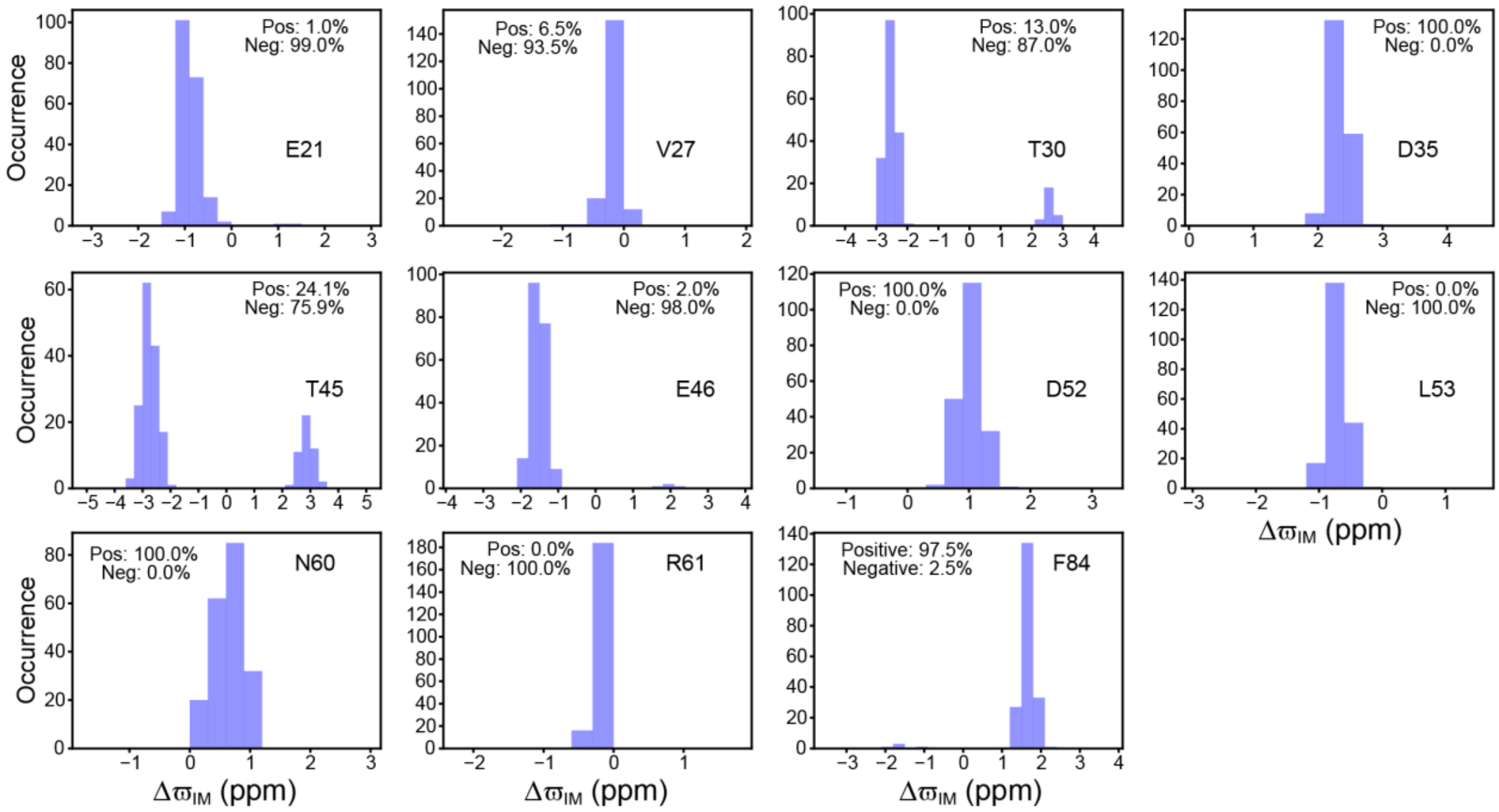

Figure S5. Bootstrap-based assignment of the sign of $\Delta\varpi_{IM}$ for the remaining 11 residues of set19. For each residue, 200 bootstrapped trials were analyzed using the three-state exchange model. In each trial, the global parameters p_I, p_M, $k_{ex,FI}$, $k_{ex,FM}$, and $k_{ex,IM}$ were fixed to one of the values obtained from the 200 bootstrap analyses of set8 residues, whereas the residue-specific parameters $\Delta\varpi_{FI}$, $\Delta\varpi_{IM}$, R_{1F} and R_{2F} were fitted independently. Constraints R_{2M}=R_{2I}=R_{2F}, and R_{1M}=R_{1I}=R_{1F} were used. Because the sign of $\Delta\varpi_{IM}$ could not be assigned directly from the two-state analysis for these residues, each bootstrap dataset was fitted twice, once with $\Delta\varpi_{IM}$>0 and once with $\Delta\varpi_{IM}$<0. The solution with the lower reduced χ^2_{red} value was retained. Histograms show the resulting distributions of $\Delta\varpi_{IM}$, and the percentages indicate how often the positive or negative solution was selected. For 10 of the 11 residues, one sign was preferred in more than 80% of the bootstrap trials, indicating a robust assignment of the relative chemical-shift sign.

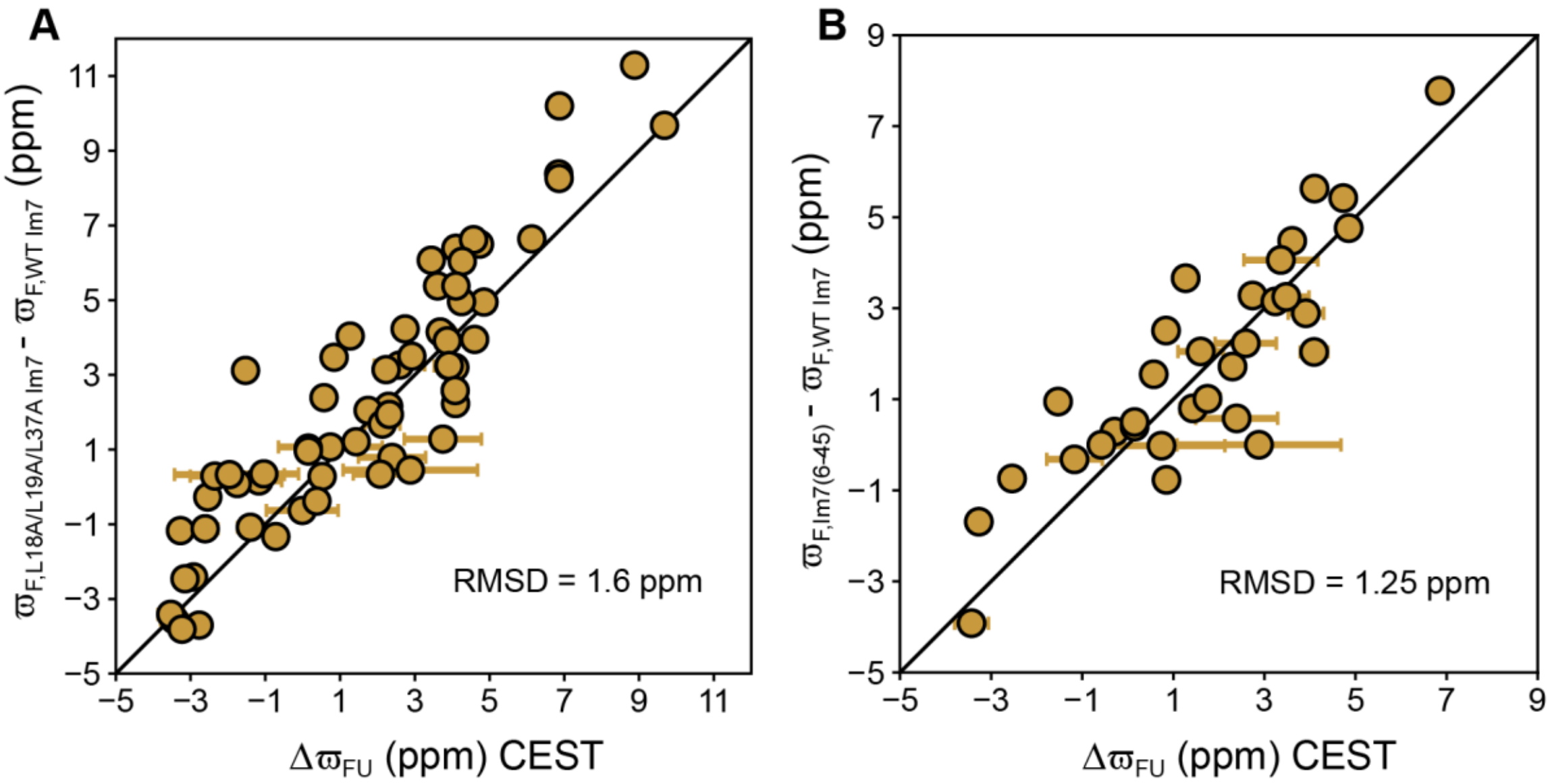

Figure S6. Comparison of the unfolded-state chemical shifts derived from multi-field CEST with previously characterized unfolded-state mimics of Im7. Residue-specific ^{15}N chemical-shift differences between the folded and unfolded states, $\Delta\varpi_{FU}$, obtained from the three-state CEST analysis plotted against the corresponding chemical-shift differences derived from the L18A/L19A/L37A Im7 variant[23] (A) and the Im7(6–45) fragment[24] (B). The root-mean-square deviations are 1.60 ppm and 1.25 ppm for panels A and B, respectively, demonstrating close agreement between the CEST-derived unfolded-state chemical shifts and those of the previously reported unfolded-state mimics.

Detectability of minor exchange by ^{15}N CEST versus ^{15}N CPMG

To establish the range of $k_{ex,IU}$ and p_U over which the unfolded state U of Im7 is detectable by ^{15}N CEST and ^{15}N CPMG experiments, synthetic ^{15}N CEST and ^{15}N TROSY-CPMG data sets were generated for a linear three-state exchange model, F↔I↔U, and each data set was then analysed using a two-state exchange model. The failure of the two-state model, quantified by the χ^2_{red} of the fit reports on whether the presence of U is encoded in the data at a level exceeding the noise. The reference parameter set was similar to those obtained from the three-state analysis of the multi-field ^{15}N CEST data of Im7: $k_{ex,FI}$ = 400 s^{-1}, $k_{ex,IU}$ = 5500 s^{-1}, p_I = 1.1 %, p_U = 0.4 %, with direct F↔U exchange set to zero ($k_{ex,FU}$ = 0). Residue-specific ϖ_F, $\Delta\varpi_{FI}$ and $\Delta\varpi_{FU}$ values for 71 residues were taken from the same three-state CEST analysis. Two sets of calculations were performed, i) where $k_{ex,IU}$ was varied from 2000 s^{-1} to 20000 s^{-1} (a total of 10 points) while keeping $k_{ex,FI}$ = 400 s^{-1}, p_I = 1.1 %, and p_U = 0.4 %; ii) where p_U was varied from 0.1% to 1.1% (a total of 8 points) while keeping $k_{ex,FI}$ = 400 s^{-1}, $k_{ex,IU}$ = 5500 s^{-1}, and p_I = 1.1 %. Noise-free profiles were first generated for each grid point with *ChemEx*. ^{15}N TROSY CPMG relaxation-dispersion profiles were simulated at ^{1}H Larmor frequencies of 600 and 800 MHz exactly as described above in "Generation of synthetic ^{15}N TROSY CPMG data" section. ^{15}N CEST profiles were simulated at a ^{1}H Larmor frequency of 800 MHz for two saturation fields, B_1 = 20 Hz with T_{ex} = 0.45 s and B_1 = 50 Hz with T_{ex} = 0.40 s, with the ^{15}N carrier at 118.0 ppm. Offsets spanned ±1200 Hz about the carrier in steps of 30 Hz (B_1= 20 Hz, 81 planes) and 40 Hz (B_1 = 50 Hz, 61 planes), each with a reference plane with T_{ex} = 0. Intrinsic relaxation rates were set to R_1 = 1 s^{-1} and R_2 = 15 s^{-1} for all three states. Ten independent noise replicas of CEST and CPMG data were generated for every grid point by adding Gaussian noise to the noise-free data as described before.

Two-state analysis of the synthetic data

Each of the 10 × 18 CPMG and 10 × 18 CEST synthetic data sets were fitted to a two-state exchange model. Starting values for $\Delta\varpi_{2st}$ were the position of the *apparent* minor-state resonance in the presence of I↔U exchange. For each residue and grid point this was obtained by diagonalizing the 3 × 3 Bloch–McConnell evolution matrix $i\Omega - R_2 + K$, built from the grid-point exchange rates, populations and residue-specific shifts (R_2 = 15 s^{-1} for all states), and taking imaginary part of the of the second-slowest-relaxing eigenmode at 800 MHz. This ensures that the two-state fit starts from the coalesced minor-state position that the data actually contain, so that a poor fit reflects the inadequacy of the two-state model rather than a poor initial guess. Fits of the CPMG data was performed exactly as described for the experimental

CPMG data before. Fits of the CEST data were performed with R_{2I}=R_{2F} and R_{1I}=R_{1F} constraints as usually done for experimental two-state analysis. The χ^2_{red} of the final stage was recorded for every replica, and is reported as the mean over the 10 replicas. A minor state was counted as detected when the χ^2_{red} of the two-state model exceeded the threshold at which we would, in practice, proceed to a three-state analysis of an experimental data set: χ^2_{red} > 1.3 for CPMG and χ^2_{red} > 1.2 for CEST. The two thresholds are based on our experience. Because the synthetic data carry Gaussian noise of precisely known magnitude, χ^2_{red} is a well-defined statistic here and a fixed threshold is meaningful; for experimental data, where uncertainties are less well characterised, such a criterion would be supplemented by visual inspection of the fits.

Results

The two experiments respond to the hidden U state in qualitatively different ways (Figure S7). For given $k_{ex,FI}$ = 400 s^{-1}, $k_{ex,IU}$ = 5500 s^{-1}, and p_I = 1.1 %, the χ^2_{red} of a two-state fit to the CEST data crosses the threshold of 1.2 already when $p_U \geq 0.2$ % and then rises steeply, whereas χ^2_{red} of the two-state fits of the CPMG data grows far more slowly and crosses its threshold only when $p_U \geq 0.9$ %. Three regimes follow: for when $p_U < 0.2$ % neither experiment detects U; for 0.2 % $\leq p_U \leq$ 0.8 % only CEST does; and for $p_U \geq 0.9$ % both do. For given $k_{ex,FI}$ = 400 s^{-1}, p_I = 1.1 %, and p_U = 0.4 %, the two-state χ^2_{red} values for both the experiments decreases monotonically as the I↔U exchange accelerates, but at very different rates. The two-state χ^2_{red} for the CEST data falls from ~3.8 at $k_{ex,IU}$ = 2000 s^{-1} to 1.07 ± 0.005 at 20 000 s^{-1}, crossing the threshold at ~11 000 s^{-1}. The two-state χ^2_{red} for the CPMG data falls from 1.4 at $k_{ex,IU}$ = 2000 s^{-1} to 1.0 at 20000 s^{-1}, crossing the threshold at $k_{ex,IU}$ = 3000 s^{-1}. Three regimes follow: for when $k_{ex,IU}$ > 11000 s^{-1} neither experiment detects U; for 3000 s^{-1} $\leq k_{ex,IU} \leq$ 11000 s^{-1} only CEST does; and for $k_{ex,IU}$ < 3000 s^{-1} both do. At no point in either scan is U detected by CPMG but not by CEST. Note that these exchange regimes will change with B_0. The Im7 network characterised in this work, p_U ~ 0.4 % and $k_{ex,IU}$ = 5500 s^{-1}, lies squarely in the CEST-only regime, which is why the three-state network is resolved by the CEST data but not by CPMG due to its effects on the minor-state dip observed in the CEST profiles. Two further points qualify these numbers. First, the two scans are one-dimensional sections through a two-dimensional parameter space that intersect at the Im7 values, so the quoted boundaries apply along those sections; the $k_{ex,IU}$ range over which CPMG detects U is expected to widen as p_U increases, and the p_U threshold to depend on $k_{ex,IU}$. Second, the comparison is deliberately

conservative towards CEST: the simulations use a single static field (800 MHz) with two B_1 settings, the CEST advantage established above is therefore a lower bound of what the full multi-field experiment achieves.

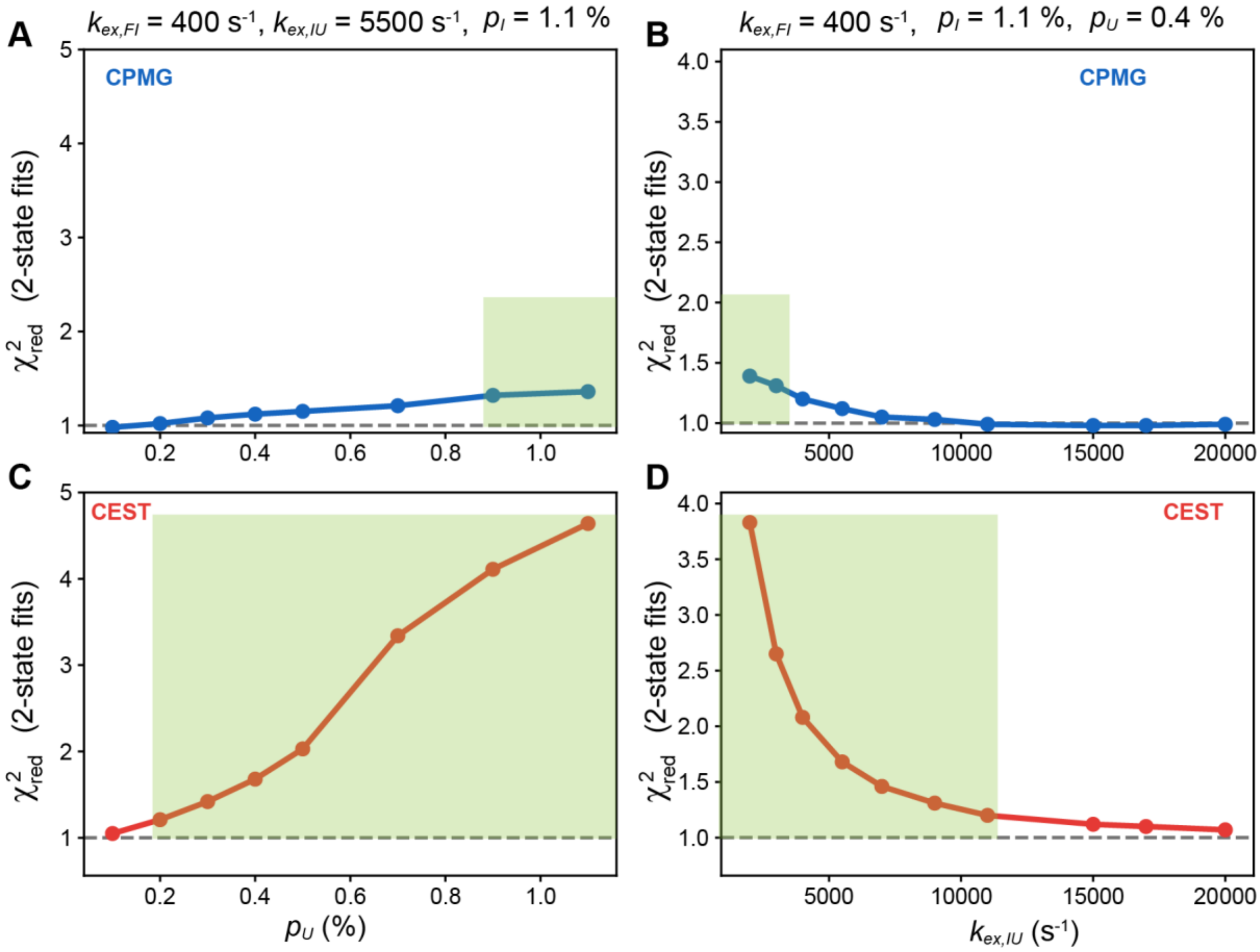

Figure S7 Detectability of minor exchange by ^{15}N CEST versus ^{15}N CPMG. χ^2_{red} values obtained from the two-state fits to synthetic data generated using a three-state F↔I↔U model is plotted. A, B) χ^2_{red} values from the fits of ^{15}N TROSY-CPMG data simulated at 600 and 800 MHz are plotted against the grid point p_U (A) and $k_{ex,IU}$ (B). C, D) χ^2_{red} values from the fits of ^{15}N CEST data simulated at 800 MHz using B_1 = 20 and 50 Hz are plotted against the grid point p_U (C) and $k_{ex,IU}$ (D). In (A, C), CPMG/CEST data was generated by varying p_U while keeping $k_{ex,FI}$ = 400 s^{-1}, $k_{ex,IU}$ = 5500 s^{-1}, and p_I = 1.1 %; in (B, D), CPMG/CEST data was generated by varying $k_{ex,IU}$ while keeping $k_{ex,FI}$ = 400 s^{-1}, p_I = 1.1 %, and p_U = 0.4 %. The dashed line indicates χ^2_{red} = 1, and the shaded regions indicate conditions where the third state is detectable according to the thresholds used (χ^2_{red} > 1.3 for CPMG and χ^2_{red} > 1.2 for CEST). Within the parameter range explored, the U state is detectable by both methods at higher populations or slower I↔U exchange, by CEST alone over an intermediate regime that includes the experimentally determined Im7 parameters, and by neither method at very low populations and rapid exchange.

Supplementary Tables

Table S1. Experimental parameters of the multi-field ^{15}N CEST experiments[a]

T (°C)	B_0 (MHz)	B_1 (Hz)	T_{EX} (ms)	Range (Hz)	Carrier (ppm)	Step-size (Hz)	CP[b]	SW[c] (ppm)
10	600	16.0	450	-900 to 900	119.228	25	42	18
		32.0	350	-900 to 900	119.228	30	42	18
	800	23.4	450	-1200 to 1215	118.229	35	48	18
		58.6	400	-1400 to 1400	118.229	40	48	18
		146.5	300	-1700 to 1700	118.229	50	48	18
		234.5	250	-2000 to 1960	118.229	60	48	18
	1200	21.8	500	-1700 to 1720	117.234	30	48	15
		54.5	450	-1900 to 1900	117.234	50	48	15
15	800	23.7	400	-1200 to 1215	118.169	35	48	18
		59.2	350	-1400 to 1400	118.169	40	48	18
20	800	23.4	400	-1200 to 1215	118.118	35	48	18
		58.5	350	-1400 to 1400	118.118	40	48	18

[a] Sample contained 1 mM [*U*-^{15}N] WT Im7 dissolved in 50 mM potassium phosphate, 0.02% (w/w) NaN_3, pH 6.6, (2% D_2O) buffer.
[b] CP stands for number of complex points in indirect dimension.
[c] SW stands for spectral-width in indirect dimension.

Table S2. Residue-specific data obtained from multi-field ^{15}N CEST.

Residue	ϖ_F (ppm)	$\Delta\varpi_{FI}$ (ppm)	Error[a] (ppm)	$\Delta\varpi_{FU}$ (ppm)	Error[a] (ppm)
T11	112.516	-0.35	0.20	2.74	0.20
E12	122.165	-1.42	0.20	1.43	0.28
A13	118.781	-0.55	0.20	3.61	0.21
E14	119.431	1.12	0.40	-1.17	0.61
F15	124.939	0.05	0.20	-3.43	0.37
V16	119.997	-1.66	0.20	-1.53	0.20
Q17	117.853	-0.22	0.20	3.36	0.81
L18	122.734	-1.31	0.20	-0.29	0.20
L19	119.324	-2.22	0.45	1.60	0.50
K20	120.830	-2.17	0.20	-0.58	0.20
E21	123.192	-2.21	0.20	-3.27	0.20
I22	120.874	-1.23	0.38	0.74	1.39
E23	118.536	1.52	0.20	4.10	0.20
K24	119.278	0.08	0.54	2.59	0.67
E25	119.308	-2.50	0.20	0.57	0.20
N26	119.301	-2.20	0.20	0.15	0.20
V27	112.765	7.00	0.20	6.85	0.20
A28	124.816	-0.39	0.20	3.91	0.39
A29	121.914	-0.39	0.20	4.09	0.30
T30	109.024	3.83	0.20	1.27	0.20
D31	121.513	-1.49	0.67	2.39	0.90
D32	118.821	0.37	0.20	2.30	0.20
V33	120.187	-0.69	0.66	2.88	1.80
L34	122.465	-2.37	0.20	0.84	0.20
D35	115.721	2.39	0.20	4.73	0.20
V36	120.506	-2.22	0.20	0.85	0.20
L37	121.018	-0.08	0.20	3.24	0.20
L38	118.889	-0.20	0.20	3.48	0.50
E39	119.904	-1.51	0.20	0.15	0.20
H40	119.814	-4.94	0.20	-2.54	0.20
F41	118.664	-3.84	0.20	2.23	0.28
V42	121.315	-3.53	0.20	1.75	0.28
K43	120.965	0.51	0.20	4.85	0.21
T45	109.429	6.70	0.20	9.68	0.20
E46	112.352	10.49	0.20	8.88	0.20
H47	119.614	-0.52	0.28	3.75	1.03
D49	121.414	-1.37	0.36	-0.01	0.96
G50	107.824	1.44	0.20	2.13	0.47
T51	114.759	-1.97	0.20	-0.72	0.20
D52	126.170	-3.87	0.20	-2.77	0.20
L53	115.579	6.91	0.20	6.13	0.20
I54	113.741	9.01	0.20	6.86	0.20
Y55	114.575	10.82	0.20	6.87	0.21
Y56	118.065	7.84	0.20	4.57	0.20
S58	117.010	0.19	0.20	-2.61	0.34

D59	121.936	0.89	0.67	-1.04	0.93
N60	115.605	2.16	0.20	2.92	0.20
R61	116.708	4.49	0.20	4.25	0.20
D62	121.030	2.85	0.20	0.52	0.20
D63	124.298	-0.21	0.20	-3.53	0.34
S64	117.516	-1.31	0.20	0.38	0.20
E66	115.834	0.07	0.20	3.76	0.36
G67	112.333	-0.15	0.20	-2.92	0.29
I68	124.498	0.24	0.20	-3.23	0.20
V69	118.924	-0.86	0.20	4.27	0.24
K70	120.177	-1.27	0.20	4.10	0.24
I72	119.280	1.53	0.20	3.04	0.20
K73	120.062	-1.00	0.20	4.07	0.24
E74	119.253	-0.52	0.30	3.44	0.26
W75	122.969	-0.14	0.23	-2.35	0.66
R76	118.731	-0.57	0.20	3.68	0.22
A77	120.285	-0.51	0.20	3.87	0.22
A78	120.687	-0.16	0.20	2.32	0.30
N79	113.489	-0.45	0.20	3.92	0.27
G80	108.281	0.03	0.87	2.08	0.73
K81	119.243	-0.53	0.20	4.08	0.32
G83	110.720	-0.34	0.20	-1.40	0.34
F84	116.369	2.89	0.20	4.60	0.20
K85	124.282	0.26	0.20	-1.75	0.29
Q86	125.504	-0.10	0.20	-3.15	0.21
G87	117.004	0.99	0.65	-1.96	1.47

[a]Errors in $\Delta\varpi$ values were estimated from 200 bootstrap analyses. A minimum uncertainty of 0.20 ppm is assumed for all $\Delta\varpi$ values.

Supplementary References

(1) He, L.; Sharpe, T.; Mazur, A.; Hiller, S. A Molecular Mechanism of Chaperone-Client Recognition. *Sci. Adv.* **2016**, *2* (11), e1601625. https://doi.org/10.1126/sciadv.1601625

(2) Tiwari, V. P.; Pandit, S.; Vallurupalli, P. Exchangeable Deuterons Introduce Artifacts in Amide 15 N CEST Experiments Used to Study Protein Conformational Exchange. *J. Biomol. NMR* **2019**, *73*, 43–48. https://doi.org/10.1007/s10858-018-00223-3

(3) Vallurupalli, P.; Bouvignies, G.; Kay, L. E. Studying "Invisible" Excited Protein States in Slow Exchange with a Major State Conformation. *J. Am. Chem. Soc.* **2012**, *134* (19), 8148–8161. https://doi.org/10.1021/ja3001419

(4) Guenneugues, M.; Berthault, P.; Desvaux, H. A Method for Determining B 1 Field Inhomogeneity. Are the Biases Assumed in Heteronuclear Relaxation Experiments Usually Underestimated? *J. Magn. Reson.* **1999**, *136* (1), 118–126. https://doi.org/10.1006/jmre.1998.1590

(5) Cui, Y.; Jin, Y.; Hou, Y.; Han, X.; Cao, H.; Kay, L. E.; Yuwen, T. Optimization of TROSY- and Anti-TROSY-Based 15N CPMG Relaxation Dispersion Experiments through Phase Cycling. *J. Magn. Reson.* **2024**, *361*, 107629. https://doi.org/10.1016/j.jmr.2024.107629

(6) Vallurupalli, P.; Hansen, D. F.; Stollar, E.; Meirovitch, E.; Kay, L. E. Measurement of Bond Vector Orientations in Invisible Excited States of Proteins. *Proc. Natl. Acad. Sci. U. S. A.* **2007**, *104* (47), 18473–18477. https://doi.org/10.1073/pnas.0708296104

(7) Loria, J. P.; Rance, M.; Palmer, A. G. A TROSY CPMG Sequence for Characterizing Chemical Exchange in Large Proteins. *J. Biomol. NMR* **1999**, *15* (2), 151–155. https://doi.org/10.1023/A:1008355631073

(8) Mulder, F. A. A.; Mittermaier, A.; Hon, B.; Dahlquist, F. W.; Kay, L. E. Studying Excited States of Proteins by NMR Spectroscopy. *Nat. Struct. Biol.* **2001**, *8* (11), 932–935. https://doi.org/10.1038/nsb1101-932

(9) Hansen, D. F.; Vallurupalli, P.; Kay, L. E. An Improved 15N Relaxation Dispersion Experiment for the Measurement of Millisecond Time-Scale Dynamics in Proteins. *J. Phys. Chem. B* **2008**, *112* (19), 5898–5904. https://doi.org/10.1021/jp074793o

(10) Vallurupalli, P.; Sekhar, A.; Yuwen, T.; Kay, L. E. Probing Conformational Dynamics in Biomolecules via Chemical Exchange Saturation Transfer: A Primer. *J. Biomol. NMR* **2017**, *67* (4), 243–271. https://doi.org/10.1007/s10858-017-0099-4

(11) Wang, A. C.; Bax, A. Minimizing the Effects of Radio-Frequency Heating in Multidimensional NMR Experiments. *J. Biomol. NMR* **1993**, *3* (6), 715–720. https://doi.org/10.1007/BF00198374

(12) Otten, R.; Villali, J.; Kern, D.; Mulder, F. A. A. Probing Microsecond Time Scale Dynamics in Proteins by Methyl 1H Carr−Purcell−Meiboom−Gill Relaxation Dispersion NMR Measurements. Application to Activation of the Signaling Protein NtrCr. *J. Am. Chem. Soc.* **2010**, *132* (47), 17004–17014. https://doi.org/10.1021/ja107410x

(13) Delaglio, F.; Grzesiek, S.; Vuister, G. W.; Zhu, G.; Pfeifer, J.; Bax, A. NMRPipe: A Multidimensional Spectral Processing System Based on UNIX Pipes. *J. Biomol. NMR* **1995**, *6* (3), 277–293. https://doi.org/10.1007/BF00197809

(14) Lee, W.; Tonelli, M.; Markley, J. L. NMRFAM-SPARKY: Enhanced Software for Biomolecular NMR Spectroscopy. *Bioinformatics* **2015**, *31* (8), 1325–1327. https://doi.org/10.1093/bioinformatics/btu830

(15) Ahlner, A.; Carlsson, M.; Jonsson, B. H.; Lundström, P. PINT: A Software for Integration of Peak Volumes and Extraction of Relaxation Rates. *J. Biomol. NMR* **2013**, *56* (3), 191–202. https://doi.org/10.1007/s10858-013-9737-7

(16) Bouvignies, G. Chemex. *(https://github.com/gbouvignies/chemex)* **2011**.
(17) Nielsen, J. T.; Mulder, F. A. A. POTENCI: Prediction of Temperature, Neighbor and pH-Corrected Chemical Shifts for Intrinsically Disordered Proteins. *J. Biomol. NMR* **2018**, *70*, 141–165. https://doi.org/10.1007/s10858-018-0166-5
(18) Estrada, J.; Bernadó, P.; Blackledge, M.; Sancho, J. ProtSA: A Web Application for Calculating Sequence Specific Protein Solvent Accessibilities in the Unfolded Ensemble. *BMC Bioinformatics* **2009**, *10*, 1–8. https://doi.org/10.1186/1471-2105-10-104
(19) Tiwari, V. P.; Toyama, Y.; De, D.; Kay, L. E.; Vallurupalli, P. The A39G FF Domain Folds on a Volcano-Shaped Free Energy Surface via Separate Pathways. *Proc. Natl. Acad. Sci.* **2021**, *118* (46), e2115113118. https://doi.org/10.1073/pnas.2115113118
(20) Kay, L.; Torchia, D.; Bax, A. Backbone Dynamics of Proteins as Studied by 15N Inverse Detected Heteronuclear NMR Spectroscopy: Application to Staphylococcal Nuclease. *Biochemistry* **1989**, *28*, 8972–8979.
(21) Gu, Y.; Hansen, A. L.; Peng, Y.; Brüschweiler, R. Rapid Determination of Fast Protein Dynamics from NMR Chemical Exchange Saturation Transfer Data. *Angew. Chem. Int. Ed.* **2016**, *55* (9), 3117–3119. https://doi.org/10.1002/anie.201511711
(22) Palmer, A. G., III. NMR Characterization of the Dynamics of Biomacromolecules. *Chem. Rev.* **2004**, *104* (8), 3623–3640. https://doi.org/10.1021/cr030413t
(23) Pashley, C. L.; Morgan, G. J.; Kalverda, A. P.; Thompson, G. S.; Kleanthous, C.; Radford, S. E. Conformational Properties of the Unfolded State of Im7 in Nondenaturing Conditions. *J. Mol. Biol.* **2012**, *416* (2), 300–318. https://doi.org/10.1016/j.jmb.2011.12.041
(24) Salmon, L.; Ahlstrom, L. S.; Horowitz, S.; Dickson, A.; Brooks, C. L., III; Bardwell, J. C. A. Capturing a Dynamic Chaperone–Substrate Interaction Using NMR-Informed Molecular Modeling. *J. Am. Chem. Soc.* **2016**, *138* (31), 9826–9839. https://doi.org/10.1021/jacs.6b02382
